# The Modified Imitation Game: A Method for Measuring Interactional Expertise

**DOI:** 10.3389/fpsyg.2021.730985

**Published:** 2021-10-29

**Authors:** Güler Arsal, Joel Suss, Paul Ward, Vivian Ta, Ryan Ringer, David W. Eccles

**Affiliations:** ^1^Envision Research Institute, Envision, Inc., Wichita, KS, United States; ^2^Department of Psychology, Fairmount College of Liberal Arts and Sciences, Wichita State University, Wichita, KS, United States; ^3^Social and Behavioral Sciences, The MITRE Corporation, McLean, VA, United States; ^4^Department of Psychology, Lake Forest College, Lake Forest, IL, United States; ^5^Department of Educational Psychology and Learning Systems, College of Education, Florida State University, Tallahassee, FL, United States

**Keywords:** blindness, contributory expertise, imitation game, interactional expertise, natural-language processing, signal detection, tacit knowledge

## Abstract

The study of the sociology of scientific knowledge distinguishes between contributory and interactional experts. Contributory experts have practical expertise—they can “walk the walk.” Interactional experts have internalized the tacit components of expertise—they can “talk the talk” but are not able to reliably “walk the walk.” Interactional expertise permits effective communication between contributory experts and others (e.g., laypeople), which in turn facilitates working jointly toward shared goals. Interactional expertise is attained through long-term immersion into the expert community in question. To assess interactional expertise, researchers developed the imitation game—a variant of the Turing test—to test whether a person, or a particular group, possesses interactional expertise of another. The imitation game, which has been used mainly in sociology to study the social nature of knowledge, may also be a useful tool for researchers who focus on cognitive aspects of expertise. In this paper, we introduce a modified version of the imitation game and apply it to examine interactional expertise in the context of blindness. Specifically, we examined blind and sighted individuals’ ability to imitate each other in a street-crossing scenario. In Phase I, blind and sighted individuals provided verbal reports of their thought processes associated with crossing a street—once while imitating the other group (i.e., as a pretender) and once responding genuinely (i.e., as a non-pretender). In Phase II, transcriptions of the reports were judged as either genuine or imitated responses by a different set of blind and sighted participants, who also provided the reasoning for their decisions. The judges comprised blind individuals, sighted orientation-and-mobility specialists, and sighted individuals with infrequent socialization with blind individuals. Decision data were analyzed using probit mixed models for signal-detection-theory indices. Reasoning data were analyzed using natural-language-processing (NLP) techniques. The results revealed evidence that interactional expertise (i.e., relevant tacit knowledge) can be acquired by immersion in the group that possesses and produces the expert knowledge. The modified imitation game can be a useful research tool for measuring interactional expertise within a community of practice and evaluating practitioners’ understanding of true experts.

## Introduction

In the last half century, there has been a shift in perspective from one that viewed cognition as taking place in the head using mental representations driven exclusively by symbols (e.g., Vera and Simon, 1993) to one that recognizes cognition as being crucially dependent on active situated interaction with the world (e.g., Hutchins, 1995). Numerous theoretical perspectives exploring the relationship between action and performance (e.g., distributed cognition; Hutchins, 1995; embedded cognition; Rupert, 2009; situated cognition; Brown et al., 1989) have highlighted the importance of cognition in context. For instance, from a situated perspective, knowledge is dynamically constructed and socially reproduced, and its acquisition cannot be separated from the context in which it is acquired (Lave and Wenger, 1991). The primary concern of these perspectives relates to understanding the experience of expert practitioners using methods that involve the dynamic interaction between humans and their environment (Baber, 2020).

In cognitive accounts of expertise, knowledge that is difficult to express in propositional form (i.e., tacit knowledge) is the basis for developing intuitive reasoning and decision-making skills (Klein, 1998). Tacit knowledge is deeply rooted in action and context, and its acquisition requires considerable experience obtained in operational settings. Sociological accounts of expertise (Collins and Evans, 2020) also attach importance to tacit knowledge and emphasize the acquisition of expertise through socialization (Collins, 2010).

### Sociological Accounts of Expertise

In the study of the sociology of scientific knowledge, Collins and Evans (2007, 2020) have developed a sociological perspective on expertise and a framework for classifying different levels of expertise and knowledge. According to this perspective, domain expertise is the property of individuals and groups, and one’s level of expertise grows as a result of being embedded within a society of experts. In Collins and Evans’s (2007, 2020) classification of expertise, the most substantive expertise requires specialist tacit knowledge that cannot be gained without deep social immersion in the groups that possess and produce the expert knowledge. Collins and Evans (2007, 2020) described two kinds of specialist tacit knowledge: contributory expertise and interactional expertise.

The concept of contributory expertise is comparable to the conventional, cognitive account of expertise, which defines expertise in terms of individual accomplishment and practice (e.g., deliberate practice; Ericsson et al., 1993). A contributory expert could be described as someone who performs at a reliably superior level and adaptively engages in skilled practice within a specific domain (e.g., Ward et al., 2018, 2020). An interactional expert, on the other hand, is someone who does not have the ability to perform a skilled practice, *per se*, but is exposed to the tacit knowledge of the domain through full socialization or immersion in an expert community (Collins and Evans, 2007). In short, for interactional experts, tacit knowledge pertains to the language of the domain, not its practice.

Interactional expertise permits effective communication between true experts and others, such as laypeople or novices, to work jointly toward shared goals. Given that today’s complex societal and environmental problems require interdisciplinary teams, interactional experts can facilitate communication within these teams, and integrate different forms of knowledge. For example, scientists aiming to address important problems, such as climate change, must integrate knowledge and skills from across disciplines. Similarly, engineers deliberating about what users want and need, must step outside their disciplines and incorporate knowledge produced in the other fields and contexts. Therefore, interactional experts are an important agent of information dissemination between groups with disparate knowledge pools and goal orientations.

Two important conditions for within-group communication are (1) whether members of interdisciplinary teams have learned the language used by others in the domain by interacting with true experts, and (2) if they have acquired important tacit knowledge in the process. In this paper, we focus on individuals who lack the practical competence in a specialized domain and assess whether they have acquired the tacit knowledge of that domain through immersion. Specifically, our aim is to determine whether one group (e.g., sighted individuals) has acquired interactional expertise of the other (e.g., how blind individuals cross streets), and vice versa.

In the next sections, we first explain a research method known as the imitation game, which was developed to measure interactional expertise (Collins and Evans, 2014; Collins et al., 2017). Second, we present modifications to the method that make it suitable for investigating cognitive aspects of expertise and its relation to context. Third, we report on a study that uses the modified imitation game to examine interactional expertise in the context of blindness.

### Imitation Game

The imitation game is a Turing-like test (Turing, 1950) to measure whether one person—or a particular group—possesses interactional expertise of another (Collins and Evans, 2014; Collins et al., 2017). It was designed to study the social nature of knowledge. Researchers have used the imitation game to study interactional expertise in cultural and social domains such as gender (Evans et al., 2019), national identity (Kubiak and Weinel, 2016), religiosity (Arminen et al., 2019), and sexuality (Collins et al., 2017). Other researchers have used the imitation game to explore the ability of healthcare professionals to understand the experiences of their patients (Wehrens and Walters, 2018) and discussed the potential of the imitation game as a professional training tool for medical practitioners (Evans and Crocker, 2013).

**FIGURE 1:**
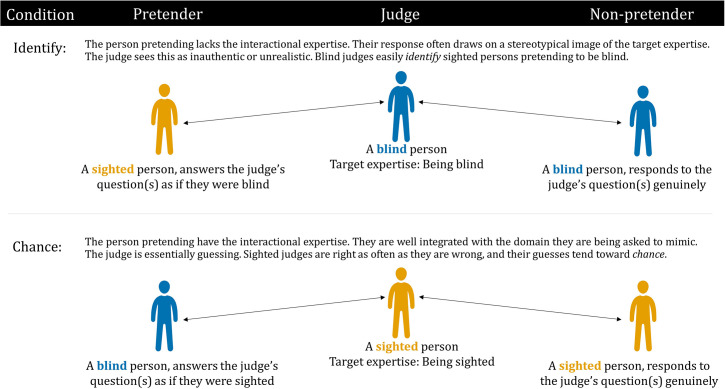
Design of Collins and Evans’s (2014) imitation game.

One previous study has used the imitation game to measure interactional expertise in the context of blindness. Collins and Evans (2014) deployed the imitation game using groups of three: a judge, a non-pretender, and a pretender (see Figure 1). The three participants in each group were seated separately and were unaware of each other’s identity or role. The judge’s task was to pose questions that would enable them to identify which participant was the non-pretender and which was the pretender. The questions were posed and answered *via* text message. There were two conditions: *identify* and *chance*. In the identify condition, both the judge and non-pretender were blind, and the pretender was a sighted person imitating a blind person. In the chance condition, both the judge and non-pretender were sighted, and the pretender was a blind person imitating a sighted person.

Collins and Evans (2014) predicted that if the pretenders possessed a high degree of interactional expertise of the other group, they should be able to imitate well and thus the judges would be unable to distinguish them from non-pretenders. The data included the questions asked by the judge, the answers provided by the pretender and non-pretender, and the judge’s decision, level of decision confidence, and reasoning about their decision. Analysis of the decision data showed that blind participants were more successful at imitating sighted people than sighted participants were at imitating blind people. One explanation for this finding was that blind people are immersed in the language of sighted people all their lives, and thus develop interactional expertise of being sighted. In contrast, sighted people do not pick up the interactional expertise of being blind, because typically they have little interaction with the blind.

The central concept of Collins and Evans’s (2014) research was socialness and, therefore, their emphasis was on the social nature of knowledge. As such, the imitation game is perfectly suited to answering questions about the social nature of knowledge, without any need for modification. But aspects of the game—its general structure, for example—also make it attractive to researchers who study the underlying cognitive mechanisms of domain-specific expertise. Here, we present some modifications to prior instantiations of the imitation game that make it more suitable for use in cognitive research (cf. research focused on the social nature of knowledge). These modifications are not responses to perceived limitations of the original imitation game; they merely represent our attempt to adapt the imitation game for a different fundamental purpose.

First, in Collins and Evans’s (2014) study, after asking a question, a judge received answers from a pretender and non-pretender concurrently and made a comparative judgment. In other words, a judge had a two-alternative forced-choice task, where strong evidence in favor of one stimulus is not necessarily evidence against the other stimulus, given that the other stimulus could be weak or strong. If non-pretenders—who are assumed to be the contributory experts—cannot generate “ideal” answers to judges’ questions, then the relative strength difference between the two presented stimuli will be less noticeable. In these instances, judges may respond less accurately as there is a smaller difference between the two stimuli. For cognitive research, this is a potential confound. Our modification involves presenting only one answer (a single stimulus) at a time and constraining the judges to make their decision based solely on the answer they are evaluating and then deciding which of two categories the stimulus belongs to.

Second, one of the central features of the original imitation game is that judges compose their own questions. This is central to the notion of interactional expertise. Judges can ask as many questions as they like, but typically ask 6–8 questions (Collins et al., 2017). It is reasonable to assume that some judges tend to pose more difficult and discriminating questions than other judges. When questions with varying difficulty levels are relayed to different pretender/non-pretender pairs, the answers will be shaped accordingly. In other words, question difficulty can be a confounding factor in the pretender’s and non-pretender’s answer. Our modification consists of including a single question posed by the researchers. In contrast to the original imitation game—in which interaction was a central feature—this modification results in only a minimal level of interaction between the judge and pretender/non-pretender.

Third, in Collins and Evans’s (2014) study, participants could assume multiple roles (e.g., judge, non-pretender) during iterative rounds of the game. Therefore, some participants could have, for example, gained knowledge as a non-pretender in one group that they then used when they later assumed the role of judge in another group. This could, theoretically, be controlled using counterbalancing. We, however, elected for a different approach: restricting participants to one role (e.g., judge) and presenting them with multiple opportunities to judge a set of standardized stimuli from pretenders and non-pretenders (i.e., a repeated-measures design).

Fourth, in Collins and Evans’s (2014) study, each judge only received one of the two possible combinations of pretender and non-pretender stimuli. For example, blind judges were always matched with a sighted pretender and a blind non-pretender (see identify condition in Figure 1); blind judges were never matched with a sighted non-pretender and a blind pretender. This made sense for the purpose the study because Collins and Evans (2014) were primarily interested in judges who are contributory experts. However, this arrangement precludes the assessment of a blind judge’s ability to distinguish between a sighted non-pretender and a blind pretender (and a sighted judge’s ability to distinguish between a sighted pretender and a blind non-pretender). Therefore, we decided to present judges with multiple trials that cover both combinations (i.e., sighted pretender/blind non-pretender, sighted non-pretender/blind pretender).

Finally, Collins and Evans’s (2014) method and data analysis ignored dependencies in the data. Specifically, some participants served in multiple roles—and thereby provided multiple data points—yet the data were treated as being independent. Although this type of analysis may have sufficed for the purpose of Collins and Evans’s (2014) study about the social nature of knowledge, cognitive psychologists often use repeated-measures designs. Consequently, they apply statistical methods that account for correlated observations in the data.

### Modified Imitation Game

We modified Collins and Evans’s (2014) imitation game so that we could focus on cognitive aspects of expertise. We made the following modifications: (1) rather than having three participants play the game together, we first elicited verbal reports from pretenders and non-pretenders by presenting them with a standardized scenario; (2) this, in effect, constrained the judges by preventing them from asking their own questions; (3) we then presented judges with the transcribed verbal reports—one by one—and asked them to decide if each report was provided by a pretender or a non-pretender; and (4) we included reports from sighted pretenders and sighted non-pretenders, as well as from blind pretenders and non-pretenders.

The modified imitation game involves two sequential phases. In Phase I, *actors* provide verbal reports of their thought processes associated with a single question composed by the researchers. In Phase II, judges make decisions about whether a verbal report obtained from an actor is from a pretender or non-pretender. As in the original version of the imitation game, there are two types of conditions: identify and chance. In the identify condition, the target expertise (i.e., the expertise that the pretender is trying to imitate) is being blind, and thus non-pretenders are blind and pretenders are sighted individuals. In the chance condition, the target expertise is being sighted, and thus non-pretenders are sighted and pretenders are blind individuals.

**FIGURE 2:**
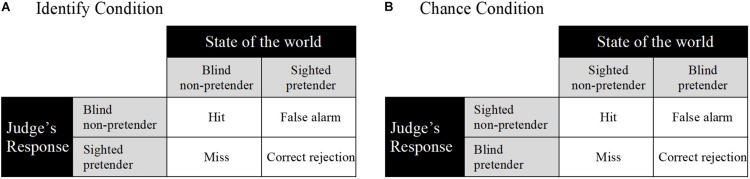
Modified imitation game and signal detection matrices for the Identify condition **(A)** and the Chance condition **(B)**. Hit: correctly indicating a signal is present; false alarm: incorrectly indicating that a signal is present; miss: incorrectly indicating that no signal is present; correct rejection: correctly indicating that no signal is present.

We argue that our modified imitation game has important benefits in terms of measurement. Specifically, the modified imitation game yields data that are amenable to an established quantitative analytic approach: signal-detection theory (Green and Swets, 1966). Judges’ binary decision-making behavior in the imitation game (see Figure 2) can be analyzed using two conceptually separate measures of performance. The first measure is sensitivity (*d*′), which is an indicator of an observer’s ability to make a binary distinction between *signal* (i.e., non-pretender) and *noise* (i.e., pretender). A *d*′ value of zero indicates chance-level performance; a positive value indicates performance better than chance and a negative value worse than chance. The second measure is response bias (β or criterion *c*), which is an indicator of an observer’s tendency to favor one of the response alternatives. A *c* value of zero indicates unbiased responding. A positive value indicates a conservative response bias; that is, a tendency to respond “pretender.” A negative value indicates a liberal response bias; that is, a tendency to respond “non-pretender.” Traditional signal-detection analysis aggregates data from each participant’s trials to calculate a hit rate and false alarm rate (Green and Swets, 1966). More recent approaches, based on generalized linear mixed models (GLMMs), use trial-level data to model fixed and random effects in categorical response data (e.g., DeCarlo, 1998, 2010, 2011; Rabe, 2018). We apply this approach in the current study.

### The Current Study

Using the modified imitation game, we wanted to determine whether one group (sighted individuals) has acquired interactional expertise of the other (how blind individuals cross streets), and vice versa. In line with Collins and Evans’s (2014) findings, we hypothesized that sighted pretenders would not be successful at pretending to be blind (Hypothesis 1). This, in turn, should make it relatively easy for contributory experts to discriminate sighted pretenders from blind non-pretenders. In other words, blind judges should be able to use their experience of being blind to discriminate sighted pretenders from blind non-pretenders. Therefore, blind judges’ (i.e., contributory experts’) sensitivity in the identify condition should be above chance-level performance (Hypothesis 1a). Additionally, if sighted pretenders cannot successfully pretend to be blind, it is reasonable to expect that even non-experts can discriminate sighted pretenders from blind non-pretenders—at least to some degree. Therefore, if sighted judges’ sensitivity in the identify condition is above chance-level performance, it should be significantly lower than that of blind judges (Hypothesis 1b).

Also consistent with Collins and Evans’s (2014) findings, we hypothesized that blind pretenders would be successful at pretending to be sighted (i.e., Hypothesis 2). This, in turn, should make it relatively difficult for contributory experts to discriminate blind pretenders from sighted non-pretenders. Therefore, sighted judges’ (i.e., contributory experts’) sensitivity in the chance condition should be at chance-level performance (Hypothesis 2a). Additionally, if blind pretenders can successfully pretend to be sighted, it is reasonable to expect that it will also be difficult for non-experts to discriminate blind pretenders from sighted non-pretenders. Therefore, blind judges’ sensitivity in the chance condition should also be at chance-level performance (Hypothesis 2b).

Up until this point, our hypotheses have focused on using judges to measure pretenders’ imitation capacity. But we are also interested in using the modified imitation game to assess the professional development of sighted orientation-and-mobility (O&M) specialists: individuals who work in the education and rehabilitation of the blind. As sighted O&M specialists are immersed in the social and cultural environment of blind individuals, we hypothesized that their sensitivity in the identify condition should be better than that of other sighted individuals (i.e., who have only infrequent social interaction with the blind). Therefore, sighted O&M specialists’ sensitivity in the identify condition should be significantly higher than that of sighted judges (Hypothesis 1c). A corresponding hypothesis for the chance condition is that sighted O&M specialists’ sensitivity in the chance condition should be at chance-level performance (Hypothesis 2c).

Last, we analyzed the judges’ textual responses regarding their rationale for each decision using natural-language-processing (NLP) techniques. Because we did not have any specific hypotheses, we explored the textual data for any meaningful patterns between groups that could provide insight into the reasons behind the judges’ decisions.

## Materials and Methods

### Phase I: Eliciting Actors’ Thought Processes

#### Participants

The Phase I participants (i.e., actors) represent a subset of participants who completed a previous study (Arsal et al., in press). That study included 36 participants (15 blind or visually impaired individuals, 15 sighted individuals, and 6 certified sighted O&M specialists). We selected eight blind and eight sighted participants’ data from that study to be used in phase I of the current study. The blind participants were selected based on their previous visual experience (i.e., those who had no, or minimal, visual experience). The sighted participants were selected at random from the group of 15 sighted participants.

The blind group comprised eight participants (four females and four males; *M*_age_ = 46.88 years, *SD* = 15.13) who were recruited through an organization for the visually impaired and paid $25 for their participation. Their degree of visual impairment varied: one with light perception, two with light projection, and five with hand movement or 20/400 visual acuity in Snellen fraction. Seven were congenitally blind (i.e., blind from birth) and one was blinded at the age of 3. Four used a long cane as their primary mobility aid; the remaining four used a guide dog. All those in the blind group had no other disability.

The sighted group comprised eight participants (four females and four males; *M*_age_ = 26.75 years, *SD* = 6.61) who were recruited *via* a university participant pool and through flyers posted on notice boards around a university campus. They were deemed eligible to participate if they responded “No” to the following screening question: “Do you consider that you have frequent social interaction with persons who are blind or visually impaired?” Seven were undergraduate students who received academic credit for participating; one was a community member who was paid $25 for their participation.

#### Task

Participants were first instructed to imagine being in an urban street-crossing scenario using the following script: “You are standing at a cross intersection where two roads intersect at right angles. On both roads, traffic flows in two directions, and the intersection is controlled by traffic lights. Your goal is to cross the road in a safe and efficient manner.” The script intentionally omitted details such as how many lanes of traffic there were in each direction, type of traffic control system (e.g., pedestrian push buttons, audible walk signals), and traffic density. Participants were then asked to think aloud while they imagined approaching the intersection and then crossing the road. As they did so, the researcher reminded them to verbalize the decisions they would make and the specific cues they would use to support these decisions. Beyond providing this level of detail, participants verbalized freely; there were no limits (i.e., minimum or maximum) on the duration of verbalization.

Using the same script, participants completed the task twice: once as a non-pretender and once as a pretender. In the non-pretender role, they verbalized their thinking naturally; that is, in line with their actual state of sightedness. In the pretender role, the blind group pretended that they did not have any visual impairments, whereas the sighted group pretended that they were blind. Participants were given instructions to aid their pretending. Specifically, the blind participants were instructed that their goal was to convince an imaginary third person—who they were to imagine was sitting in the room, blindfolded, and therefore unaware of whether the speaker was a blind person or a sighted person—that they were sighted. The sighted participants were instructed that their goal was to convince the same imaginary third person that they were blind.

#### Procedure

The study was approved by an Institutional Review Board, and participants provided informed consent. Participants were tested individually. They first completed a demographic questionnaire, with assistance from a researcher, as necessary. Next, a researcher verbally described the street-crossing scenario using the script (see section “Task”). The blind group first completed the think-aloud description as a non-pretender, and then as a pretender. The sighted group first completed the think-aloud procedure as a pretender, and then as a non-pretender. Participants’ verbalizations were audio recorded. Each testing session lasted approximately 30 min.

#### Data Processing

Participants’ verbalizations were transcribed with minor alterations for clarity, such as removing fillers and repetitions. The transcription process yielded four types of descriptions: blind non-pretender, sighted pretender, blind pretender, and sighted non-pretender. An example of each description type is provided in Table 1.

**TABLE 1:** Sample descriptions of street crossing.

**Blind non-pretender:** “When I am approaching an intersection, I am already listening for the flow of traffic. I might want to walk a little faster or slower. If the cars parallel to me have started going already, I may slow down, because I will not have enough time to cross the street. If the cars in front of me are crossing as I am approaching, then I think, ‘Okay, I want to get there so that I have a maximum amount of time.’ In that case, I will walk a little faster. Sometimes that means if there is a long wait, then I will not cross that street, but cross another street. This is an important part of the analysis–to understand what is the most efficient way to cross. There may be a rounded curb or a straight curb. After I come up to the curb, I go to the inside of the street. Because then I make sure I am in the crosswalk. In addition, because the curb is then going to straighten out as it hits the alignment of the street, and that way my dog and I can line up straight to cross the street. Next, I will look for the button to push. They are usually inconsistent, not in the same places. Sometimes you have to feel all over the place. During all of this, I am also analyzing the traffic. ‘Is there a left-hand turning lane or not?’ What I want eventually is that the cars that are in the lane close to me are going straight. That means they are protecting me from any left turning cars that might turn in front of me. If I am on the right side of the street, there may be right turning cars. Then I have to be careful, because there is no control for those people. In that case, I will exaggerate my action when I start to cross. If I am with my dog, I will wave my hand forward and making it really clear that, ‘okay, I am crossing now.’ I am kind of indicating to the world, ‘hold up.’ There may be some lines that I can feel or see little bit on crosswalks. Sometimes streets have no clear lines, but have brick, very low contrast bricks in crosswalks. Another rule with the dog is once you commit to crossing, even if the light changes, just keep going, because people stopped for you. If you are going forward and turn it around, then somebody might try to go behind you. Then you are endangering yourself! Unless it is just right after you step off the curb and the light changes” (Phase I participant B-04). **Sighted pretender:** “The first thing I will do is to listen for the traffic sound to understand what direction the traffic is coming from. Is it perpendicular to where I am trying to go or parallel? If it is perpendicular, then I will, of course, wait. I will find the crosswalk button mounted to stoplight poles. At some of the crosswalks, there are audio countdown devices that tell you how many seconds are left before the light turning red. At some crosswalks, there is only the beeping of the timer. They do not provide the verbal count down. If I come up to the intersection, and it is already beeping, I would not go. I know that time is not on my side there. I will just wait. I just wait for the next go around. If I do not hear the beep and then I start to hear it, I will know that I will have enough time to cross. Because I think, it gives you like 15 or 20 s, or something like that. I will also listen for the parallel traffic. Is it sounding like it is slowing down, or turning? At crosswalks, I may sometimes feel, with my cane, the lines, or the physical markers” (Phase I participant S-05). **Blind pretender:** “I am walking up to this intersection. I am looking around and watching people in cars and different things. The main thing I am paying attention to is the walk sign. Does the walk sign indicate that I should go or not? That is the binary thing that I am noting. Unless there is a right turning car in front of me. Then I will make eye contact with them. They will have the idea that I am going to go first. Pedestrian should have the right of way, and so I go” (Phase I participant B-04). **Sighted non-pretender:** “As I am approaching the crosswalk, even before I press the button, the first thing I would do is to see who has the right-of-way. I will look what direction traffic is going. I will check who has the green light. I generally press the push-button no matter what. Sometimes even though the light is red, you can still cross if it is safe to do so. However, typically I will just wait for the light to turn green. I usually double check before I go to see the cars are not coming” (Phase I participant S-02).

#### Descriptions

There was a total of 32 descriptions: 2 (group: blind, sighted) × 2 (role: non-pretender, pretender) × 8 actors. Blind non-pretender descriptions were longer in length (*M* = 338.00 words, *SD* = 127.41) than the sighted pretender descriptions (*M* = 184.13 words, *SD* = 45.10). The difference in length between the two types of descriptions (*M* = 153.88 words, *SE* = 47.78, 95% CI [45.26, 262.49]) was statistically significant, *t*_(__8.73)_ = 3.22, *p* = 0.001, Hedges’ *g* = 1.52, 95% CI [0.37, 2.67]. Sighted non-pretender descriptions were longer in length (*M* = 84.75 words, *SD* = 27.36) than blind pretender descriptions (*M* = 76.50 words, *SD* = 34.61). The difference in length between the two types of descriptions (*M* = 8.25 words, *SE* = 15.60, 95% CI [−25.37, 41.87]) was not statistically significant, *t*_(__13.29)_ = 0.53, *p* = 0.606, Hedges’ *g* = 0.25, 95% CI [−0.77, 1.27].

### Phase II: Judges’ Decision Making

#### Participants

We recruited three groups of participants as judges: blind, sighted, and sighted certified O&M specialists. The blind group comprised 46 participants (31 females and 15 males; *M*_age_ = 56.20 years, *SD* = 14.48) who were recruited through listserv emails sent from associations and organizations for blind and visually impaired. Blind judges were paid $25 for their participation. Their degree of visual impairment varied: 12 with total blindness, 5 with light perception, 4 with light projection, and 25 with hand movement or 20/400 visual acuity in Snellen fraction. Twenty-four were congenitally blind and 22 were blinded after birth at age 14.23, on average (*SD* = 14.24, *Mdn* = 8.5). Twenty-eight blind participants used a long cane as their primary mobility aid; the remaining 18 used a guide dog.

The sighted group comprised 136 participants (91 females, 43 males, and 2 others; *M*_age_ = 20.85 years, *SD* = 4.90) who were recruited *via* a university participant pool and received academic credit for their participation. They were deemed eligible to participate if they responded “No” to the following screening question: “Do you consider that you have frequent social interaction with persons who are blind or visually impaired?”

The O&M group comprised 39 sighted specialists (32 females and 7 males; *M*_age_ = 44.67 years, *SD* = 11.99). They were certified professionals with master’s degrees in orientation and mobility. Their experience in this profession ranged from 1 to 35 years (*M* = 13.21, *SD* = 10.06, *Mdn* = 13). They were recruited through announcements and flyers posted at a meeting of an orientation and mobility association and were paid $25 for their participation.

#### Task

Judges read or listened to descriptions and decided whether each one was elicited from a non-pretender (i.e., a genuine response) or a pretender (i.e., an imitated response). In the identify condition, judges were given the following question and response options: “Is this a blind person giving a genuine response? yes (this is a blind person) or no (this is a sighted person pretending to be blind).” In the chance condition, judges were given the following question and response options: “Is this a sighted person giving a genuine response? yes (this is a sighted person) or no (this is a blind person pretending to be sighted).” After deciding, judges rated how confident they were in their decision using an 11-point Likert-type scale ranging from 0 (*not at all confident*) to 10 (*completely confident*). After rating their confidence, judges provided a rationale for their decision, such as the factors they thought differentiated a genuine response from an imitated response.

#### Procedure

The experimental task was created as an online Qualtrics survey.^1^ Eligible participants were provided with the study web link. The link directed them to the survey, where they provided informed consent and completed a demographic questionnaire including items on vision impairment and O&M teaching experience. Next, participants received general instructions about the task (see Supplementary Material). Subsequently, they undertook the task, which was presented in 2 blocks of 16 trials. One block comprised identify trials, in which judges decided whether each description was elicited from a blind non-pretender (8 trials) or a sighted pretender (8 trials). The other block comprised chance trials, in which judges decided whether each description was elicited from a sighted non-pretender (8 trials) or a blind pretender (8 trials). In each block, participants were first given condition-specific instructions (see Supplementary Material). The order of the blocks was randomized, as were the order of trials within each block. Blind judges used screen-reader software to transform the text that displayed on the computer screen to synthesized speech or braille display.

## Data Analysis and Results

Judges’ decision and reasoning data were analyzed with GLMMs using R 4.0.2 (R Core Team, 2020) and the lme4 package (v1.1-23; Bates et al., 2015). The reproducible code and data for these models can be accessed *via* Supplementary Material. We followed Meteyard and Davies (2020) guidelines for building models, evaluating effects, and reporting results.

### Judges’ Decisions

#### Data Analysis

We specified a binomial distribution with a probit link function to assess signal-detection indices at a single-trial level of analysis (DeCarlo, 1998). This contrasts with standard signal-detection-theory analysis, which involves aggregating trial information to obtain indices (e.g., hits, misses, false alarms, and correct rejections) for each participant. GLMMs are also organized in a nested hierarchy, allowing for tests of both main effects and interactions of population-level (i.e., fixed) effects. The criterion variable was the probability of responding “yes” vs. “no” in a given trial (coded as 1 and 0, respectively). Sensitivity (*d*′) was calculated by including *Type* (pretender vs. non-pretender, coded as −0.5 and 0.5, respectively) as the first predictor variable in the model. Thus, the slope for Type indicates the difference in response probability between pretender and non-pretender trials, and subsequent interactions with Type indicate changes in sensitivity. Conversely, parameter estimates in which Type is absent reflect response bias. *Condition* (chance and identify) and *Group* (blind, O&M, and sighted) were also included as fixed effects. Because trial-level information was preserved, we were able to account for random variability among *Participants* (i.e., by-subject random effects), *Descriptions*, and *Actors* (i.e., by-item random effects). Simple models include only random intercepts (i.e., they only account for individual variability in participants’ overall response biases), while random-slope models can account for individual variability across all within-subjects variables, but at the cost of additional effects degrees of freedom. Thus, optimal model fit was determined in a two-step process (Bates et al., 2015): the first step established the optimal random-effects structure, and the second step determined the optimal fixed-effects structure.

To establish the optimal random-effects structure, we first generated a *maximal model* in which the fixed-effects structure included a three-way interaction between all predictor variables (e.g., Type, Condition, and Group) and fully crossed by-subject random effects with an additional interaction term for response confidence. Description and Actor were included as by-item random intercepts. This model failed to converge and resulted in a singular fit, suggesting that it was overparameterized in its effects structure. Therefore, Principal Components Analysis (PCA) was performed on the random effects to determine the optimal random-effects structure using the rePCA() function in the lme4 package. This analysis (see Supplementary Material) found the by-subject random effects of Intercept, Type, and Condition accounted for 55.9, 29.0, and 9.9% of the residual variance, respectively, or 94.8% of the cumulative total. Thus, the optimal by-subject random-effects structure included random intercepts, as well as random slopes of Type and Condition.

In the second step of model selection, the fixed-effects structures were varied step-wise to determine the optimal model fit. Five models were generated (see Table 2 for model specifications) and model fitness was evaluated using likelihood ratio tests (Bates et al., 2015). First, we specified a baseline model that included only the intercept as its fixed effect for predicting the *Response* (non-pretender or pretender) without any additional predictors. Model 2 included the predictor Type (non-pretender description vs. pretender description) as a fixed effect to determine if participants were at all sensitive to the task. We then sequentially included additional predictors as fixed effects, namely, Condition (chance, identify) and Group (blind, O&M, and sighted).

**TABLE 2:** Model specifications in R package lme4.

Model	Fixed-effects structure
Model 1	Response ∼ 1
Model 2	Response ∼ Type
Model 3	Response ∼ Type × Condition
Model 4	Response ∼ Type × Group
Model 5	Response ∼ Type × Condition × Group

*Response indicates judges’ responses. Type indicates whether the description was non-pretender on a given trial.*

#### Results

Table 3 shows the results of model comparisons. Model 5 had the lowest akaike information criterion (AIC) (8720.2) and highest −2LL (−4340.1) and chi-square (34.84) values. The likelihood ratio tests showed that Model 5 was the best-fitting model. Therefore, response bias and sensitivity results are reported based on the estimates of Model 5 (see Table 4).

**TABLE 3:** Model comparisons.

							χ^2^		
Model	*df*	AIC	BIC	ΔAIC	ΔBIC	−2LL	Value	*df*	*P*
Model 1	9	8751.5	8813.3			−4366.8			
Model 2	10	8741.0	8809.6	–10.5	–3.7	−4360.5	12.52	1	< 0.001*
Model 3	12	8744.8	8827.2	3.8	17.6	−4360.4	0.17	2	0.919
Model 4	14	8743.0	8839.1	–1.8	11.9	−4357.5	5.78	2	0.056
Model 5	20	8720.2	8857.5	–22.8	18.4	−4340.1	34.84	6	< 0.001*

ΔAIC, ΔBIC, and χ^2^ values indicate the change in goodness of fit between each subsequent model. *p < 0.05.

**TABLE 4:** The estimates of Model 5.

Random effects	Variance	*SD*	*r*	
Participant (Intercept)	0.06	0.25		
Type	0.08	0.29	–0.16	
Condition	0.01	0.09	0.45	0.37
Description (Intercept)	0.15	0.39		
Actor (Intercept)	0.00	0.00		

**Fixed effects**	**Estimate**	***SE***	***Z***	***P***

(Intercept)	0.299	0.074	4.03	< 0.001*
Type	0.561	0.145	3.88	< 0.001*
Condition	–0.035	0.072	–0.49	0.624
Group (Blind)	0.097	0.040	2.41	0.016*
Group (O&M)	–0.060	0.042	–1.42	0.156
Group (Sighted)	–0.038	0.032	–1.18	0.238
Type × Condition	–0.124	0.143	–0.87	0.384
Type × Group (Blind)	0.030	0.065	0.45	0.651
Type × Group (O&M)	–0.023	0.068	–0.34	0.735
Type × Group (Sighted)	–0.007	0.052	–0.13	0.897
Condition × Group (Blind)	–0.044	0.030	–1.45	0.146
Condition × Group (O&M)	0.025	0.031	–0.82	0.412
Condition × Group (Sighted)	0.018	0.024	0.77	0.442
Type × Condition × Group (Blind)	–0.222	0.056	–3.95	< 0.001*
Type × Condition × Group (O&M)	–0.016	0.058	–0.27	0.788
Type × Condition × Group (Sighted)	0.238	0.044	5.35	< 0.001*

The summary report used maximum likelihood estimation (Laplace approximation) with a binomial probit link function. The coding system used compares the mean of the dependent variable for a given level to the overall mean of the dependent variable. *p < 0.05.

##### Response bias

**FIGURE 3:**
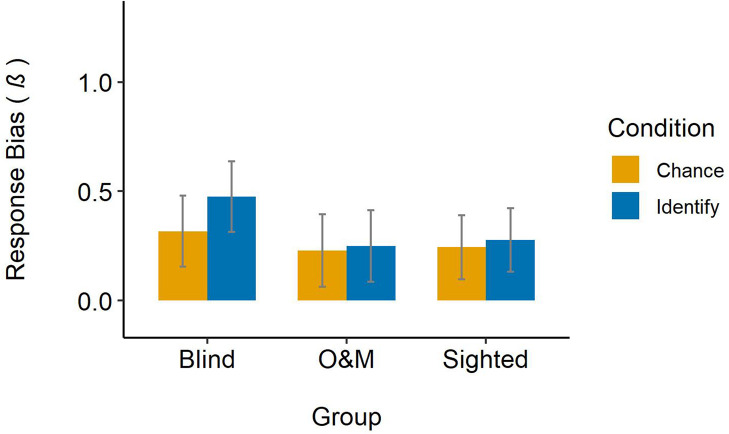
Judges’ response bias across groups and conditions. Error bars represent 95% confidence intervals.

The intercept of the model reflects any overall bias in answering yes/no (criterion *c* = −1 × intercept). Overall, the judges had a liberal response bias, β = 0.299, *z* = 4.03, *p* < 0.001. In other words, judges tended to report that descriptions were from a non-pretender than a pretender, regardless of condition or group. Judges’ response bias in the chance condition (β = 0.264), was not significantly different from the identify condition (β = 0.334), *z* = −0.49, *p* = 0.624. The blind judges’ response bias (β = 0.396) was significantly higher (i.e., more liberal) than the grand mean (β = 0.299), *z* = 2.41, *p* = 0.016. The response biases of the O&M (β = 0.239) and sighted (β = 0.261) judges were not significantly different from the grand mean (β = 0.299), *z* = −1.42, *p* = 0.156 and *z* = −1.18, *p* = 0.238, respectively. Figure 3 displays judges’ response bias across groups and conditions.

##### Sensitivity

**FIGURE 4:**
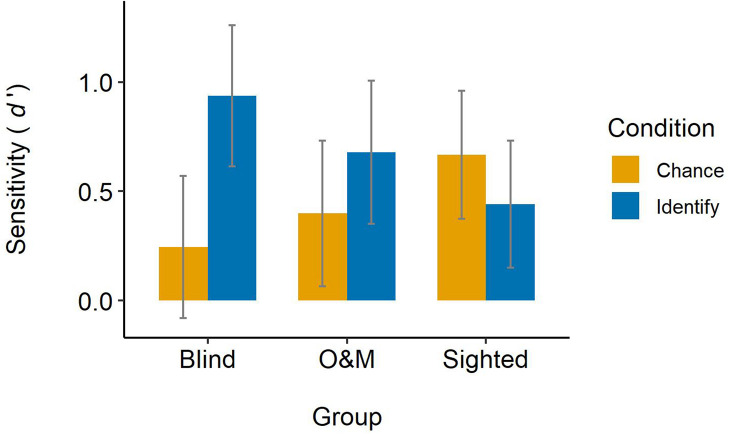
Judges’ sensitivity across groups and conditions. Error bars represent 95% confidence intervals.

Figure 4 displays judges’ sensitivity across groups and conditions. Hypothesis 1a was that blind judges’ sensitivity in the identify condition should be above chance-level performance. In the identify condition, the blind group’s sensitivity was *d*′ = 0.937, 95% CI [0.613, 1.261]. As the confidence interval does not contain 0, there is evidence that the blind judges’ sensitivity in the identify condition is indeed above chance-level performance. Hypothesis 1b was that if sighted judges’ sensitivity in the identify condition is above chance-level performance, it should still be significantly lower than that of blind judges. In the identify condition, the sighted judges’ sensitivity was *d*′ = 0.441, 95% CI [0.150, 0.733], which is above chance-level performance. Importantly, the results of the pairwise contrasts showed that the sighted judges’ sensitivity in the identify condition (*d*′ = 0.441) was indeed significantly lower than that of blind judges (*d*′ = 0.937), *z* = 3.81, *p* < 0.001. Hypothesis 1c was that sighted O&M specialists’ sensitivity in the identify condition should be significantly higher than that of sighted judges. In the identify condition, the O&M judges’ sensitivity was *d*′ = 0.678, 95% CI [0.351, 1.005], which is above chance-level performance. However, the results of the pairwise contrasts showed that the O&M judges’ sensitivity in the identify condition (*d*′ = 0.678) was not significantly higher than that of sighted judges (*d*′ = 0.441), *z* = 1.77, *p* = 0.076.

Hypothesis 2a was that sighted judges’ sensitivity in the chance condition should be at chance-level performance. In the chance condition, the sighted judges’ sensitivity was *d*′ = 0.668, 95% CI [0.374, 0.961], which is above chance-level performance. Hypothesis 2b was that blind judges’ sensitivity in the chance condition should be at chance-level performance. In the chance condition, the blind judges’ sensitivity was *d*′ = 0.244, 95% CI [−0.081, 0.569]. As this confidence interval contains 0, there is evidence that the blind judges’ sensitivity in the chance condition is at chance-level performance. Hypothesis 2c was that O&M judges’ sensitivity in the chance condition would be at chance-level performance. In the chance condition, the O&M judges’ sensitivity was *d*′ = 0.398, 95% CI [0.066, 0.731], which is above chance-level performance.

### Judges’ Reasoning

#### Data Pre-processing

Judges’ reasoning about their decision included textual data, which was analyzed using an exploratory approach to look for patterns. We were interested in judges’ reasoning when they made correct decisions only. Of the 7,072 total trials (221 judges × 32 trials), judges made 4,206 correct decisions. The textual data of these correct decisions was first pre-processed and cleaned by transforming all text to lower case, removing punctuation, and correcting typographical errors using the NLP (version 0.2-0; Hornik, 2018) and quanteda (version 2.0.1; Benoit et al., 2018) packages in R. Responses that were empty (i.e., judges who did not provide a reason for a given correct decision) or only included “idk” (i.e., I don’t know), “n/a,” or “yes” were omitted from the analyses (*n* = 630) to ensure that only meaningful data were analyzed. The final dataset consisted of *n* = 3,576 reasons.

#### Linguistic Feature Extraction

The textual responses were submitted into the software tool Linguistic Inquiry and Word Count (LIWC; Pennebaker et al., 2015) to extract linguistic features for each response. LIWC contains an internal lexicon (∼6,400 terms) for 92 linguistic features and extracts these features by calculating the percentage of terms that belong to a given linguistic feature category for a given corpus of text (Pennebaker et al., 2015). Although LIWC extracted all 92 linguistic features for every textual response in our dataset, we only analyzed 14 of those features—the ones we believed to be the most relevant to our goal at hand. These linguistic features included: *comparisons*, *cognitive processes*, *insight*, *causation*, *tentativeness*, *discrepancy*, *certainty*, *differentiation*, *perceptual processes*, *see*, *feel*, *hear*, *body*, and *space*. *Cognitive processes* is an aggregate measure of the features pertaining to cognitive processes (i.e., *insight*, *causation*, *tentativeness*, *discrepancy*, *certainty*, and *differentiation*), and *perceptual processes* is an aggregate measure of the features pertaining to perceptual processes (i.e., *see*, *feel*, and *hear*). Table 5 provides the descriptions and examples of these features.

**TABLE 5:** Linguistic inquiry and word count (LIWC) categories examined in reasoning data.

Linguistic feature	Description	Example terms
Comparisons	Language involving the comparison of an entity with another	Bigger, worse, smaller
Cognitive processes	Language reflecting human cognitive processes	Cause, identify
Insight	Language associated with gaining an accurate and deep intuitive understanding of something	Think, know
Causation	Language associated with connecting a person or thing that causes an effect	Because, effect
Tentativeness	Language reflecting something that is not fully developed or worked out	Maybe, perhaps
Discrepancy	Language reflecting a lack of similarity or agreement	Should, would
Certainty	Language reflecting confidence and assuredness	Always, never
Differentiation	Language that distinguishes between entities, people, or ideas	Hasn’t, else
Perceptual processes	Language reflecting perceptual orientations	Look, heard
See	Language referring to the perceptual process of seeing	View, saw
Feel	Language referring to the perceptual process of feeling	Feels, touch
Hear	Language referring to the perceptual process of hearing	Listen, hearing
Body	Language referring to the body	Feet, hands
Space	Language referring to the position of two or more items relative to one another	Down, in

#### Data Analysis

Type, Condition, and Group were entered as fixed effects, and Participants, Descriptions, and Actors were entered as random effects. We also examined the three-way interaction between Type, Condition, and Group. The 14 linguistic features were entered as the dependent variable in separate GLMMs, with each model predicting a separate linguistic feature. The results of these exploratory analyses can be accessed *via* Supplementary Material. In the next sections, we highlight the results of the following two linguistic features: comparisons and certainty. We believe these two models provide the most consistent and meaningful insights into the reasons underlying the judges’ decisions, and thus might provide a promising avenue for further investigation in follow-up studies.

#### Comparisons

In the identify condition, the blind judges’ reasoning for the non-pretender descriptions (*M* = 0.210, *SE* = 0.072) contained (a) significantly more comparison terms—language involving the comparison of an entity with another—than for the pretender descriptions (*M* = −0.176, *SE* = 0.085, *p* < 0.001) and (b) significantly more comparison terms than those of the sighted (*M* = −0.053, *SE* = 0.053, *p* < 0.001) and O&M groups (*M* = −0.236, *SE* = 0.096, *p* < 0.001). In the chance condition, the blind judges’ reasoning for the non-pretender descriptions (*M* = −0.036, *SE* = 0.078) contained significantly less comparison terms than for the pretender descriptions (*M* = 0.242, *SE* = 0.090, *p* = 0.015). Similarly, in the chance condition, both the sighted and O&M judges’ reasoning for the non-pretender descriptions (*M* = −0.034, *SE* = 0.054; *M* = −0.126, *SE* = 0.104, respectively) contained significantly less comparison terms than for the pretender descriptions (*M* = 0.123, *SE* = 0.058, *p* = 0.042; *M* = 0.220, *SE* = 0.115, *p* = 0.019, respectively).

We further explored the chance-condition data to better understand why limited comparison terms were found in all groups for the non-pretender rather than the pretender descriptions. We came upon a possible interpretation: The judges’ reasoning suggested that the non-pretender descriptions conveyed specific information which made it relatively apparent that they were obtained from sighted individuals. As a result, the judges in all the groups seemed to simply point to this information as their rationale. It is possible that in this condition the non-pretender descriptions did not provide as many opportunities for judges to elaborate on their rationale and make comparisons compared to the pretender descriptions. A more detailed report of this analysis is provided in Supplementary Material.

#### Certainty

In the identify condition, the blind judges’ reasoning for the non-pretender descriptions (*M* = 0.376, *SE* = 0.070) contained (a) significantly more certainty terms—language reflecting confidence and assuredness—than for the pretender descriptions (*M* = 0.125, *SE* = 0.083, *p* = 0.006) and (b) significantly more certainty terms than those of the sighted (*M* = −0.057, *SE* = 0.044, *p* < 0.001) and O&M groups (*M* = 0.076, *SE* = 0.097, *p* = 0.013). In the chance condition, the sighted judges’ reasoning for the non-pretender descriptions (*M* = −0.001, *SE* = 0.045) contained significantly more certainty terms than for the pretender descriptions (*M* = −0.149, *SE* = 0.049, *p* = 0.009).

## Discussion

In this study, we used a modified version of the imitation game to test two main hypotheses. The first hypothesis was that sighted individuals who have infrequent social interaction with blind or visually impaired individuals would not be successful at pretending to be blind. This hypothesis was supported by the data. Specifically, the blind judges could reliably discriminate between the blind non-pretenders and sighted pretenders. Unlike blind individuals, sighted individuals are not contributory experts in the domain of navigation without vision, as they have not experienced embodied practice in this domain directly. However, even the sighted groups in our study (i.e., the sighted and sighted O&M judges) could reliably discriminate between the blind non-pretenders and sighted pretenders. It appears that the verbal reports elicited from the sighted pretenders did not depict the distinctive set of experiences of blind individuals’ street crossing behavior. Therefore, we argue that sighted individuals, who have infrequent social interaction with blind or visually impaired individuals, do not have the interactional expertise needed to pass, linguistically, as a member of the blind group or culture. These findings support the notion that the acquisition of the relevant tacit knowledge can be acquired only by immersion in the society of those who already possess it (Collins, 2010).

The present study brings forth two additional findings related to the task of distinguishing the blind non-pretenders from the sighted pretenders. First, as hypothesized, the sighted judges’ discrimination ability was significantly lower than that of blind judges. This finding provides some evidence that sighted individuals are less fluent than blind individuals in the linguistic and cultural repertoires of blind communities. Second, contrary to our expectation, the sighted O&M judges did not differ from sighted judges in their discrimination ability. Although sighted O&M specialists have explicit knowledge in this domain and are assumed to have more social interactions with the blind community than other sighted individuals, their insights into the knowledge of blind individuals appears not to be superior to those of other sighted individuals. However, it is important to note that, in this study, we did not test the extent to which sighted O&M specialists have developed interactional expertise in the experiential knowledge of blind individuals. Future studies that include O&M specialists as pretending actors in Phase I of the modified imitation game—and using their verbal reports as stimuli in Phase II of the game—would reveal whether O&M specialists can demonstrate the linguistic fluency in the practice-language of blind groups.

The second hypothesis was that blind individuals would be successful at pretending to be sighted. However, this prediction, which was based on the idea that blind people are immersed in the language of sighted people all their lives, and thus easily develop interactional expertise of being sighted (Collins and Evans, 2014), was not supported by the data. Specifically, both the sighted and sighted O&M judges could reliably discriminate between the sighted non-pretenders and blind pretenders. It appears that the verbal reports elicited from the blind pretenders did not depict the distinctive set of experiences of sighted individuals’ street-crossing behavior. Therefore, we argue that blind individuals do not have the interactional expertise needed to pass, linguistically, as a member of the sighted group or culture.

One possible explanation for why blind people in the study were not successful at pretending to be sighted stems from the Dreyfus–Collins debate about the connection between expertise and embodiment (Collins, 1996, 2000, 2017; Dreyfus, 1996; Selinger, 2003). According to Dreyfus (1996) and other phenomenologists, embodiment is central not only to our experience of the world but also to our ability to understand it and the concepts we use to do so. This strong perspective on embodiment holds that if a person cannot carry out an activity themselves, then they cannot possibly understand it. From this perspective, it is not surprising that blind people were not successful at pretending to be sighted, as they have not crossed the street as a sighted person. In contrast, Collins advocates for the minimal embodiment thesis, which allows that through the accumulation of interactional expertise, a person can gain the ability to understand worlds that they cannot inhabit themselves. But even though many blind people are immersed in the language of sighted people and could therefore develop international expertise in some aspects of the sighted world, they face an additional barrier if they have never had the experience of seeing. In other words, sighted people can experience what it is like—to some extent—to be blind by closing their eyes (or stumbling around in the dark). But blind people, especially those who are congenitally blind, will never have had the experience of seeing and therefore may not have been able to reasonably answer questions about what it is like to see.

An additional finding, related to the task of distinguishing the sighted non-pretenders from the blind pretenders, was that the blind judges could not distinguish between the two. Further studies are needed to understand the reasons why the blind pretenders were not able to convince sighted individuals (i.e., contributory experts in the domain of navigation with vision) but were able to convince the blind individuals (i.e., not contributory experts in the domain of navigation with vision). It would be advantageous for future investigations to recruit blind pretenders with different levels of socialization to sighted individuals and test whether those pretenders’ knowledge about the practices related to this specific domain is enough to produce an authentic account of sighted individuals’ actual street-crossing behavior.

The imitation game generates qualitative data in the form of reasons provided by judges. In our investigation, we aimed to understand the reasons given by judges for their decisions by employing an exploratory approach. Specifically, we focused on identifying any meaningful patterns in judges’ reasoning for correct decisions by applying NLP techniques. After extracting several linguistic features from the reasons, we focused our analysis on two linguistic features: comparisons and certainty. The results showed that when correctly identifying the blind non-pretenders (i.e., the non-pretender descriptions obtained from blind individuals), the blind judges were thinking, in depth, of their own personal experiences of being blind. We assume that they reflected on their experiences for any similarities or differences, thus signaling a commitment to their judgments. On the other hand, when correctly identifying the blind non-pretenders, the sighted groups (both the sighted judges and sighted O&M judges) appeared to not to use comparisons and certainty terms as much as the blind judges. The results also showed that less comparison terms were used by the three groups of judges when correctly identifying the sighted non-pretenders (i.e., the non-pretender descriptions obtained from sighted individuals) than when correctly identifying the blind pretenders (i.e., the pretender descriptions obtained from blind individuals). It seems that the sighted non-pretender descriptions conveyed specific information which made it relatively apparent that the individual was sighted.

We have identified three main limitations to this study. First, there is a lack of counterbalanced order of the non-pretender and pretender roles across the actor groups in Phase I. An order effect may have been introduced due to the procedure of the blind actors first completing the non-pretender role, and then the pretender role, whereas the sighted actors first completing the pretender role, and then the non-pretender role. Second, only one screening question was used to determine whether the sighted actors in Phase I and the non-O&M sighted judges in Phase II had social interactions with blind persons. Recall that these individuals were deemed eligible for participation based on a screening question of “Do you consider that you have frequent social interaction with persons who are blind or visually impaired?” with a binary response (0 = no; 1 = yes). This question focuses simply on the frequency of interactions and does not consider the complexities of groups’ relationships and different forms of social interaction and sociability. A better operational definition and rigorous measurement of social interaction should have been explored. For example, future studies may include additional questions concerning actions, practices, and subjective experiences of these groups. Third, an *a priori* power analysis was not performed to determine sample size. There was no preliminary data available to estimate an effect size during the planning of the study.

It is also important to mention that there are two major limitations of the modified imitation game. These limitations stem from the fact that the judges’ role in the modified imitation game is substantially restricted compared to the original imitation game. First, unlike the original imitation game, judges in the modified game do not invent the question(s) themselves, do not work out what kinds of questions to ask, and do not have the option of asking a new question or ending the game. More importantly, judges in the original imitation game are free to ask about any topic, but they typically try to come up with questions that reveal information they believe will be known by members of the expert community in question, but not outsiders. As a matter of fact, researchers have shown that analyzing the range of question types reveals significant sociologically issues, such as categorizations of social groups or differing ways in which cultural divides are expressed (Arminen et al., 2019). Unfortunately, this kind of analysis cannot be implemented using the modified imitation game. The second limitation is that in the modified imitation game, the verbal reports obtained from pretenders and non-pretenders are isolated from further context (beyond that provided in the report itself) and do not allow for the recognition-primed questions-and-answers (Klein, 1993) that are part of the original imitation game. Judges of the original imitation game are more likely to distinguish pretenders from non-pretenders because the answers may tap implicit knowledge that they may use subsequently to pose follow-up questions.

A possible strength of the study lies in the fact that we formulated our signal-detection model as a mixed effects logistic regression model (DeCarlo, 1998) rather than standard signal-detection analysis. Our approach considers the binomial distribution of the data and allows for modeling possible random effects arising from individual differences between subjects and experimental items. Another advantage of multilevel models is that they can also address continuous stimuli and responses. Although the stimuli and response types in our study were binary (e.g., “Yes, this is a blind person” or “No, this is a sighted person pretending to be blind”), future studies of the modified imitation game can benefit from employing continuous stimuli and responses using fuzzy signal-detection theory (Parasuraman et al., 2000; Szalma and Hancock, 2013).

## Conclusion

Although lacking practical competence in a domain of expertise, individuals with interactional expertise possess the knowledge necessary to communicate with true experts at an expert level. Interactional expertise is acquired through enculturation processes, long-term immersion, and linguistic exposure into the expert community in question. The imitation game is a valuable tool for assessing the presence of interactional expertise. In this paper, several modifications were introduced to adapt the imitation game method as a research tool for those interested in the cognitive aspects of expertise. Using the modified imitation game, we assessed whether one group (e.g., sighted individuals) was indistinguishable from contributory experts (e.g., blind individuals) in conversation, and vice versa.

Using the modified imitation game, researchers can determine whether the members of one group are able to take the perspective of others and articulate their lived experiences in a domain in ways that go beyond everyday understandings. If these individuals are fully immersed in the contributory experts’ “way of life,” they should be able to speak the language of their discipline and pass as contributory experts in the imitation game. Given that interactional expertise enables different communities to understand each other and supports the efficacy of interdisciplinary teams, it is important to develop ways to measure interactional expertise in interdisciplinary teams. Individuals lacking the embodied or direct experience can strive to become interactional experts by observing the way the contributory experts behave and by spending significant amounts of time with them. Across time, these individuals may internalize the discourse and judgments of those experts and insiders, thereby developing interactional expertise. This is important, for example, in the design of usable and accessible technology such as navigation technology and accessible pedestrian signals. Typically, designers of such technology are sighted individuals who may have little interactional expertise. Finding efficient ways to increase interactional expertise could result in better—and more usable—designs. The modified imitation game potentially provides a way to measure the development of interactional expertise.

## Data Availability Statement

The datasets presented in this study can be found in online repositories. The names of the repository/repositories and accession number(s) can be found in the article/Supplementary Material.

## Ethics Statement

The studies involving human participants were reviewed and approved by the Wichita State University Institutional Review Board. The participants provided their written informed consent to participate in this study.

## Author Contributions

GA, JS, and PW were involved in planning and contributed to the design and implementation of the research. GA collected the experimental data. GA, JS, and RR performed the data analysis related to the judges’ decision. GA and VT performed the data analysis related to the judges’ reasoning. JS, PW, and DE aided in interpreting the results. All authors discussed the results and contributed to writing the manuscript.

## Author Disclaimer

PW’s affiliation with the MITRE Corporation is provided for identification purposes only and is not intended to convey or imply MITRE’s concurrence with, or support for, the positions, opinions, or viewpoints expressed by the author.

©2021 The MITRE Corporation. All rights reserved. Approved for public release. Distribution unlimited 20-03298-1.

## Conflict of Interest

GA was employed by Envision Research Institute, Envision, Inc. PW was employed by the MITRE Corporation. The remaining authors declare that the research was conducted in the absence of any commercial or financial relationships that could be construed as a potential conflict of interest.

## Publisher’s Note

All claims expressed in this article are solely those of the authors and do not necessarily represent those of their affiliated organizations, or those of the publisher, the editors and the reviewers. Any product that may be evaluated in this article, or claim that may be made by its manufacturer, is not guaranteed or endorsed by the publisher.

## Footnotes

1
www.qualtics.com

## Supplementary Material

Supplementary Table 1 Instructions for Procedure – Phase II.docx.

Supplementary Table 2 Reproducible Code for Judges Decision.docx.

Supplementary Table 3 Principal Components Analysis.docx.

Supplementary Table 4 Reproducible Code for Judges Reasoning.docx.

Supplementary Table 5 Exploratory Analyses for Reasoning Data.docx.

Supplementary Presentation 1 Description of Supplementary Material files.

Supplementary Data Sheet 1 Demographic_Phase_I.csv.

Supplementary Data Sheet 2 Demographic_Phase_II.csv.

Supplementary Data Sheet 3 Judges_Decision_Data.csv.

Supplementary Data Sheet 4 Judges_Reasoning_Data.csv.
