# Supplementary material for: The Modified Imitation Game: A Method for Measuring Interactional Expertise
Source: Front Psychol. 2021 Oct 29;12:730985. doi: 10.3389/fpsyg.2021.730985 (PMC8586539; doi:10.3389/fpsyg.2021.730985)
Supplement: Supplementary Table 1 — Instructions for Procedure – Phase II.docx. [file Table_1.docx]

**Instructions for Procedure - Phase II**

**General Instructions**

Your task will be to read/listen to a text where an individual is talking about street crossing techniques and strategies. After reading/listening to the paragraph, you will make a decision about the individual. There are several texts obtained from several individuals. We asked these individuals to imagine themselves in the following urban street-crossing scenario: “You are standing at a cross intersection where two roads intersect at right angles. On both roads, traffic flows in two directions and the intersection is controlled by traffic lights. Your goal is to cross the road in a safe and efficient manner.” Afterwards, we asked these individuals to think aloud while they imagined themselves approaching the intersection and then crossing the road. There were two groups of individuals: blind group and sighted group. The individuals in the blind group had severe or more profound vision impairment. The individuals in the sighted group had normal vision, and reported infrequent social interaction with blind or visually impaired individuals.

**Instructions for Condition without Vision (Identify Condition)**

In the next section, you will be asked to read/listen to a text where an individual is talking about navigating without vision. In these texts, the blind group is verbalizing their thinking naturally, whereas the sighted group is pretending that they are blind. In other words, the blind individuals are providing a genuine description of their street crossing techniques, whereas the normally sighted individuals are imitating and putting themselves in shoes of blind individuals. Now, we are asking you to read/listen to these texts one by one and decide whether they are from a blind person or sighted person.

**Instructions for Condition with Vision (Chance Condition)**

In the next section, you will be asked to read/listen to a text where an individual is talking about navigating with vision. In these texts, the sighted group is verbalizing their thinking naturally, whereas the blind group is pretending that they are sighted. In other words, the sighted individuals are providing a genuine description of their street crossing techniques, whereas the blind individuals are imitating and putting themselves in shoes of sighted individuals. Now, we are asking you to read/listen to these texts one by one and decide whether they are from a sighted person or blind person.
