# Supplementary material for: The Modified Imitation Game: A Method for Measuring Interactional Expertise
Source: Front Psychol. 2021 Oct 29;12:730985. doi: 10.3389/fpsyg.2021.730985 (PMC8586539; doi:10.3389/fpsyg.2021.730985)
Supplement: Supplementary Table 2 — Reproducible Code for Judges Decision.docx. [file Table_2.docx]

**Reproducible Code for Judges Decision**

To run this code in R, select the code on the following pages, copy it, and then paste into a blank Rmarkdown (RMD) file.

---

title: "Analysis of Judges’ Decisions"

author: "Guler Arsal"

date: "4/30/2021"

output: html_document

---

$~$

# Import Packages

```{r}

library(tidyverse)

library(lme4)

library(ggplot2)

library(lattice)

library(MASS)

library(ggpubr)

library(emmeans)

```

$~$

# Import Data File

```{r}

mydat <- read.csv("Judges_Decision_Data.csv")

summary(mydat)

```

$~$

# Notes About the Variables in the Data File

1. P_Id: Participant (i.e., judges) identification number(n=221)

2. Group: A between-subject factor with 3 levels: (1) Blind, (2) OM (Sighted Orientation & Mobility specialists), and (3) Sighted (Control)

3. Actor_id: Actor identification code (n=16)

4. Description: 32 descriptions elicited from actors (textual)

5. Type: It indicates whether the trial was non-pretender (coded as 1) or pretender (coded as 0)

6. Condition: A within-subject factor with two levels: identify and chance

7. Response: Participants’ response (1=the description is from a non-pretender; 0=the description is from a pretender)

8. Confidence: Participants' level of decision confidence, ranging from 0 (not at all confident) to 10 (completely confident)

9. Reasoning: Participants' reasoning about their decision (textual)

$~$

# Calculate Signal Detection Indices

```{r}

mydat <- mydat %>%

mutate(hit = case_when(Response == 1 & (Type == "1") ~ 1, TRUE ~ 0),

false_alarm = case_when(Response == 1 & (Type== "0") ~ 1, TRUE ~ 0),

miss = case_when(Response == 0 & (Type == "1") ~ 1, TRUE ~ 0),

corr_rej = case_when(Response == 0 & (Type == "0") ~ 1, TRUE ~ 0))

```

$~$

# Data Preparation for Generalized Linear Mixed Modelling

```{r}

# If judges responded that the description that they read is from a non-pretender

mydat$Response = ifelse(((mydat$hit==1) | (mydat$false_alarm==1)), 1, 0)

# If the TYPE of description was non-pretender

mydat$Type = ifelse(((mydat$Type=="1")), 0.5, -0.5)

# Make CONDITION categorical & effects coding / sums to zeros

mydat$Condition = as.factor(mydat$Condition)

contrasts(mydat$Condition)=contr.sum(2)

# Make GROUP categorical & effects coding / sums to zeros

mydat$Group = as.factor(mydat$Group)

contrasts(mydat$Group)=contr.sum(3)

# Make Participant Identification Number categorical

mydat$P_id = as.factor(mydat$P_id)

# Make Description categorical

mydat$Description = as.factor(mydat$Description)

# Make Actor categorical

mydat$Actor_id = as.factor(mydat$Actor_id)

```

$~$

# Checking the Normality of "Confidence"

```{r}

densityplot(~Confidence, data=mydat) # Density plot shows that Confidence is negatively skewed

mydat$Confidence1 <- mydat$Confidence+1 # Add 1 to Confidence so that you can run box-cox test

bc1 <- boxcox(Confidence1~1, data=mydat, lambda = seq(-2,4,length = 10), plotit=TRUE) # Run box-cox test

with(bc1, x[which.max(y)]) # Lambda is 1.636364

mydat$c.conf <- mydat$Confidence-7 # Transforming Confidence

```

$~$

# Step1: Establishing the optimal random-effects structure

## Generating A Maximal Model

```{r}

# control=glmerControl(optimizer="bobyqa",optCtrl=list(maxfun=150000))

# fit.max = glmer(Response~Type*Condition*Group + (Type*Condition*c.conf|P_id) + (1|Description) + (1|Actor), data = mydat, family=binomial(link="probit"))

```

$~$

## Principal Components Analysis

```{r}

# summary(rePCA(fit.max)) # Each column in the PCA represents the by-subj random effect component for its order in the ranef() list.

# [,1] = Intercept, [,2] = Type, [,3] = Condition

```

$~$

# Step 2: Determining the Optimal Fixed-effects Structure

```{r}

fit1 = glmer(Response~1 + (Type+Condition|P_id) + (1|Description) + (1|Actor_id), data = mydat, family=binomial(link="probit"))

fit2 = glmer(Response~Type + (Type+Condition|P_id) + (1|Description) + (1|Actor_id), data = mydat, family=binomial(link="probit"))

fit3 = glmer(Response~Type*Condition + (Type+Condition|P_id) + (1|Description) + (1|Actor_id), data = mydat, family=binomial(link="probit"))

fit4 = glmer(Response~Type*Group + (Type+Condition|P_id) + (1|Description) + (1|Actor_id), data = mydat, family=binomial(link="probit"))

fit5 = glmer(Response~Type*Condition*Group + (Type+Condition|P_id) + (1|Description) + (1|Actor_id), data = mydat, family=binomial(link="probit"))

anova(fit1, fit2, fit3, fit4, fit5)

```

$~$

## Model Comparisions

```{r}

anova(fit2, fit5) # Model 5 was identified to be the best-fitting model

```

$~$

## Parameter Estimates of the "Best Fit" Model (i.e., Model 5)

```{r}

anova(fit5)

summary(fit5)

```

$~$

# Data Visualization

## Creaating Figure 3 (Judges’ Response Bias across Groups & Conditions) & Figure 4 (Judges’ Sensitivity across Groups & Conditions)

```{r}

# To plot the fixed effects, use the model to create a "model matrix" where you feed in specific values to create means & standard errors

newdat <- NULL # Create a null model called newdat. If you make a mistake anywhere in the model matrix, you will want to reset your model matrix to "null" so you don't layer your mistake with your fix.

newdat <- expand.grid(Type=c(0,1) -.5, Group=c("Blind", "OM", "Sighted"), Condition=c("chance", "identify"), Response = 0)

contrasts(newdat$Condition)=contr.sum(2) #The contrasts need to be coded for your data frame in the same way that your contrasts were coded in the model.

contrasts(newdat$Group)=contr.sum(3)

# Extracting the parameter estimates from the fixed effects.

modelToPlot<-fit5

mm <- model.matrix(terms(modelToPlot),newdat)

newdat$Response <- mm %*% fixef(modelToPlot)

pvar1 <- diag(mm %*% tcrossprod(vcov(modelToPlot),mm))

tvar1 <- pvar1+VarCorr(modelToPlot)$P_id[1]

newdat <- data.frame(

newdat

, dPrime = (newdat$Response)

, plo = ((newdat$Response)-sqrt(pvar1))

, phi = ((newdat$Response)+sqrt(pvar1)))

```

$~$

## Calculate Sensitivity (d')

```{r}

# To get d', subtract Responded(c.Valid=.5)-Responded(c.Valid=-.5) within each condition.

# To automate this process, create three new empty variables

dPrime <- c() # The recepticle for the difference between odd and even rows.

dprime_odd <- c() # The probability of a False Alarm.

dprime_even <- c() # The probability of a Hit.

# Seperates the dPrime column in newdat and places even and odd row indices into their own list

for (i in 1:nrow(newdat)){

if (i %% 2 == 0){

dprime_even <- append(dprime_even, newdat[i, 5])

}else{

dprime_odd <- append(dprime_odd, newdat[i, 5])}}

# Then use the previous lists (odd and even) to compute dPrime and append each value to its own list.

for (i in 1:6){

cal.dPrime <- dprime_even[i] - dprime_odd[i]

dPrime <- append(dPrime, cal.dPrime)}

# Create two new empty variables

dprime_phi <- c()

dprime_plo <- c()

# For phi, subtract Odd Plo from Even Phi. For plo, subtract Odd Phi from Odd Plo.

# Phi = Highest Hit Rate - Lowest False Alarm Rate. Plo = Lowest Hit Rate - Highest False Alarm Rate

for (i in 1:12){

if (i %% 2==0){

dprime_plo.subt = newdat[i, 6] - newdat[i-1, 7]

dprime_plo <- append(dprime_plo, dprime_plo.subt)

}else{

dprime_phi.subt = newdat[i+1, 7] - newdat[i, 6]

dprime_phi <- append(dprime_phi, dprime_phi.subt)}}

# Create new vectors for the group and condition

Group <- c("Blind", "O&M", "Sighted", "Blind", "O&M", "Sighted")

Condition <- c("chance", "chance", "chance", "identify", "identify", "identify")

gdprime <- data.frame(Group, Condition, dPrime,dprime_phi,dprime_plo)

```

$~$

## Create Sensitivity Figure (Figure 4)

```{r}

g1 <- ggplot(gdprime, aes(x=Group, y=dPrime, size=36, fill=Condition)) + geom_bar(mapping = NULL, stat="identity", position="dodge", width=.7, size=1.5) + ylim(-0.1,1.3)

g1 + geom_errorbar(aes(ymin = dprime_plo, ymax = dprime_phi), position=position_dodge(.7), width=.1, size=0.5, color="gray50")+xlab("\nGroup")+ylab(expression(atop("Sensitivity ("~italic("d")~"\u2032)"),paste("\n"))) + theme(text = element_text(size=36)) + theme_pubr(legend = "right") + scale_fill_manual(labels = c("Chance", "Identify"), values = c("#E69F00","#0072B2"))

width = 5

height = (3/5)*width

ggsave("Figure_Sensitivity.jpg", height=height, width=width)

```

$~$

## Caculate Response Bias (beta)

```{r}

# To automate this process, create three new empty variables

beta <- c() # The recepticle for the difference between odd and even rows.

beta_odd <- c() # The probability of a False Alarm.

beta_even <- c() # The probability of a Hit.

# Seperates the dPrime column in newdat and places even and odd row indices into their own list

for (i in 1:nrow(newdat)){

if (i %% 2 == 0){

beta_even <- append(beta_even, newdat[i, 5])

}else{

beta_odd <- append(beta_odd, newdat[i, 5])}}

# Then use the previous lists (odd and even) to compute beta and append each value to its own list.

for (i in 1:6){

cal.beta <- (beta_even[i] + beta_odd[i])/2

beta <- append(beta, cal.beta)}

beta_phi <- c()

beta_phi_even <- c()

beta_phi_odd <- c()

for (i in 1:nrow(newdat)){

if (i %% 2 == 0){

beta_phi_even <- append(beta_phi_even, newdat[i, 7])

}else{

beta_phi_odd <- append(beta_phi_odd, newdat[i, 7])}}

for (i in 1:6){

cal.beta_phi <- (beta_phi_even[i] + beta_phi_odd[i])/2

beta_phi <- append(beta_phi, cal.beta_phi)}

beta_plo <- c()

beta_plo_even <- c()

beta_plo_odd <- c()

for (i in 1:nrow(newdat)){

if (i %% 2 == 0){

beta_plo_even <- append(beta_plo_even, newdat[i, 6])

}else{

beta_plo_odd <- append(beta_plo_odd, newdat[i, 6])}}

for (i in 1:6){

cal.beta_plo <- (beta_plo_even[i] + beta_plo_odd[i])/2

beta_plo <- append(beta_plo, cal.beta_plo)}

gbeta <- data.frame(Group, Condition, beta,beta_phi,beta_plo)

```

$~$

## Create Response Bias Figure (Figure 3)

```{r}

g2 <- ggplot(gbeta, aes(x=Group, y=beta, size=36, fill=Condition)) + geom_bar(mapping = NULL, stat="identity", position="dodge", width=.7, size=1.5) + ylim(-0.1, 1.3)

g2 + geom_errorbar(aes(ymin = beta_plo, ymax = beta_phi), position=position_dodge(.7), width=.1, size=0.5, color="gray50")+xlab("\nGroup") + ylab(expression(atop("Response Bias ("~italic("\u03B2")~")"),paste("\n"))) + theme(text = element_text(size=36))+ theme_pubr(legend = "right") + scale_fill_manual(labels = c("Chance", "Identify"), values = c("#E69F00","#0072B2"))

width = 5

height = (3/5)*width

ggsave("Figure_ResponseBias.jpg", height=height, width=width)

```

$~$

# Pairwise contrasts

```{r}

emt <- emtrends(fit5, ~ Group | Condition, var = "Type")

emt # list the estimated slopes

contrast(emt, interaction = "pairwise") # all contrasts, pairwise

```
