# Supplementary material for: The Modified Imitation Game: A Method for Measuring Interactional Expertise
Source: Front Psychol. 2021 Oct 29;12:730985. doi: 10.3389/fpsyg.2021.730985 (PMC8586539; doi:10.3389/fpsyg.2021.730985)
Supplement: Supplementary Table 3 — Principal Components Analysis.docx. [file Table_3.docx]

**Principal Components Analysis**

*Principal Components Analysis of Random Effects Structure for the Maximal Model*

|  | Component | | | | | | | | |
| --- | --- | --- | --- | --- | --- | --- | --- | --- | --- |
|  | Intercept | Type | Condition | Confidence | Type  ×  Condition | Type  ×  Confidence | Condition  ×  Confidence | Type  ×  Condition  ×  Confidence |  |
| Standard Deviation | 0.3411 | 0.2455 | 0.14348 | 0.10396 | 0.0002265 | 0.0002237 | 8.32e-05 | 3.315e-06 |  |
| Proportion of Variance | 0.5593 | 0.2897 | 0.09898 | 0.05197 | 0.0000000 | 0.0000000 | 0.00e+00 | 0.000e+00 |  |
| Cumulative Proportion of Variance | 0.5593 | 0.8490 | 0.94803 | 1.00000 | 1.0000000 | 1.0000000 | 1.00e+00 | 1.000e+00 |  |

The variance of the components
