# Supplementary material for: The Modified Imitation Game: A Method for Measuring Interactional Expertise
Source: Front Psychol. 2021 Oct 29;12:730985. doi: 10.3389/fpsyg.2021.730985 (PMC8586539; doi:10.3389/fpsyg.2021.730985)
Supplement: Supplementary Table 4 — Reproducible Code for Judges Reasoning.docx. [file Table_4.docx]

**Reproducible Code for Judges Reasoning**

To run this code in R, select the code on the following pages, copy it, and then paste into a blank Rmarkdown (RMD) file.

---

title: "Untitled"

author: "Guler Arsal"

date: "4/30/2021"

output: html_document

---

$~$

# Import Packages

```{r}

library(lme4)

library(emmeans)

```

$~$

# Import Data File

```{r}

df <- read.csv("Judges_Reasoning_Data.csv")

```

$~$

# Notes About the Observations in the Data File

As explained in the manuscript, were interested in judges’ reasoning when they made correct decisions only. Of the 7,072 total trials (221 judges × 32 trials), judges made 4,206 correct decisions. The judges’ textual responses regarding their rationale for each correct decision were first pre-processed and cleaned by transforming all text to lower case, removing punctuation, and correcting typographical errors using the NLP (version 0.2-0; Hornik, 2018) and quanteda (version 2.0.1; Benoit et al., 2018) packages in R. Responses that were empty (i.e., judges who did not provide a reason for a given correct decision) or only included “idk” (i.e., I don’t know), “n/a”, or “yes” were omitted from the analyses (n = 630) to ensure that only meaningful reasoning data were analyzed. The final dataset consisted of n = 3,576 reasons.

$~$

# Notes About the Variables in the Data File

1. P_Id: Participant (i.e., judges) identification number

2. Group: A between-subject factor with 3 levels: (1) Blind, (2) OM (Sighted Orientation & Mobility specialists), and (3) Sighted (Control)

3. Actor_id: Actor identification code (n=16)

4. Description_id: 32 descriptions elicited from actors (numbers)

5. Description: 32 descriptions elicited from actors (textual)

6. Type: It indicates whether the trial was non-pretender (coded as 1) or pretender (coded as 0)

7. Condition: A within-subject factor with two levels: identify and chance

8. Response: Participants’ response (1=the description is from a non-pretender; 0=the description is from a pretender)

9. Confidence: Participants' level of decision confidence, ranging from 0 (not at all confident) to 10 (completely confident)

10. Reasoning: Participants' reasoning about their decision (textual)

11. Column 11 to Column 96 are LIWC linguistic feature categories

$~$

# Data Preparation - Contrast coding

```{r}

df$Type = as.factor(df$Type)

contrasts(df$Type) <- matrix(data = c(.5,-.5), nrow = 2, ncol = 1, dimnames = list(c("nonpretender", "pretender"), c("nonpretender"))) # Pretender is the reference group

df$Group = as.factor(df$Group)

contrasts(df$Group) <- matrix(data = c(0, 1, 0, 0, 0, 1), nrow = 3, ncol = 2, dimnames = list(c("blind", "om", "sighted"), c("om", "sighted"))) # Blind is the reference group

df$Condition = as.factor(df$Condition)

contrasts(df$Condition) <- matrix(data = c(-.5,.5), nrow = 2, ncol = 1, dimnames = list(c("chance", "identify"), c("identify"))) # Identify condition is the reference group

```

$~$

# GLMMs

## The linguistic feature: Comparisons

```{r}

compare <- lmerTest::lmer(scale(compare) ~ Type*Condition*Group + (1|P_id) + (1|Description_id) + (1| Actor_id), data = df, REML = F)

print(summary(compare),correlation = F)

```

$~$

```{r}

emm_options(disable.pbkrtest = TRUE)

emm_options(lmerTest.limit = 3576)

emmeans(compare, ~ Condition*Group*Type )

contrast(emmeans(compare, ~ Type| Condition | Group), interaction = c("pairwise"))

contrast(emmeans(compare, ~ Condition | Type | Group), interaction = c("pairwise"))

contrast(emmeans(compare, ~ Group | Type | Condition ), interaction = c("pairwise"))

```

$~$

## The linguistic feature: Cognitive processes

```{r}

cogproc <- lmerTest::lmer(scale(cogproc) ~ Type*Condition*Group + (1|P_id) + (1|Description_id) + (1|Actor_id), data = df, REML = F)

print(summary(cogproc),correlation = F)

```

$~$

## The linguistic feature: Insight

```{r}

insight <- lmerTest::lmer(scale(insight) ~ Type*Condition*Group + (1|P_id) + (1|Description_id) + (1|Actor_id), data = df, REML = F)

print(summary(insight),correlation = F)

```

$~$

## The linguistic feature: Causation

```{r}

cause <- lmerTest::lmer(scale(cause) ~ Type*Condition*Group + (1|P_id) + (1|Description_id) + (1|Actor_id), data = df, REML = F)

print(summary(cause),correlation = F)

```

$~$

## The linguistic feature: Tentativeness

```{r}

tentat <- lmerTest::lmer(scale(tentat) ~ Type*Condition*Group + (1|P_id) + (1|Description_id) + (1|Actor_id), data = df, REML = F)

print(summary(tentat),correlation = F)

```

$~$

## The linguistic feature: Discrepancy

```{r}

discrep <- lmerTest::lmer(scale(discrep) ~ Type*Condition*Group + (1|P_id) + (1|Description_id) + (1|Actor_id), data = df, REML = F)

print(summary(discrep),correlation = F)

```

$~$

## The linguistic feature: Certainty

```{r}

certain <- lmerTest::lmer(scale(certain) ~ Type*Condition*Group + (1|P_id) + (1|Description_id) + (1|Actor_id), data = df, REML = F)

print(summary(certain),correlation = F)

```

$~$

```{r}

emm_options(disable.pbkrtest = TRUE)

emm_options(lmerTest.limit = 3576)

emmeans(certain, ~ Condition*Group*Type )

contrast(emmeans(certain, ~ Type| Condition | Group), interaction = c("pairwise"))

contrast(emmeans(certain, ~ Condition | Type | Group), interaction = c("pairwise"))

contrast(emmeans(certain, ~ Group | Type | Condition ), interaction = c("pairwise"))

```

$~$

## The linguistic feature: Differentiation

```{r}

differ <- lmerTest::lmer(scale(differ) ~ Type*Condition*Group + (1|P_id) + (1|Description_id) + (1|Actor_id), data = df, REML = F)

print(summary(differ),correlation = F)

```

$~$

## The linguistic feature: Perceptual processes

```{r}

percept <- lmerTest::lmer(scale(percept) ~ Type*Condition*Group + (1|P_id) + (1|Description_id) + (1|Actor_id), data = df, REML = F)

print(summary(percept),correlation = F)

```

$~$

## The linguistic feature: See

```{r}

see <- lmerTest::lmer(scale(see) ~ Type*Condition*Group + (1|P_id) + (1|Description_id) + (1|Actor_id), data = df, REML = F)

print(summary(see),correlation = F)

```

$~$

## The linguistic feature: Feel

```{r}

feel <- lmerTest::lmer(scale(feel) ~ Type*Condition*Group + (1|P_id) + (1|Description_id) + (1|Actor_id), data = df, REML = F)

print(summary(feel),correlation = F)

```

$~$

## The linguistic feature: Hear

```{r}

hear <- lmerTest::lmer(scale(hear) ~ Type*Condition*Group + (1|P_id) + (1|Description_id) + (1|Actor_id), data = df, REML = F)

print(summary(hear),correlation = F)

```

$~$

## The linguistic feature: Body

```{r}

body <- lmerTest::lmer(scale(body) ~ Type*Condition*Group + (1|P_id) + (1|Description_id) + (1|Actor_id), data = df, REML = F)

print(summary(body),correlation = F)

```

$~$

## The linguistic feature: Space

```{r}

space <- lmerTest::lmer(scale(space) ~ Type*Condition*Group + (1|P_id) + (1|Description_id) + (1|Actor_id), data = df, REML = F)

print(summary(space),correlation = F)

```

$~$

# Word count as DV; data from Identify Condition only

```{r}

WC <- lmerTest::lmer(scale(WC) ~ Type*Group + (1|P_id) + (1|Description_id) + (1|Actor_id), data = df, REML = F)

print(summary(WC),correlation = F)

```
