# Supplementary material for: The Modified Imitation Game: A Method for Measuring Interactional Expertise
Source: Front Psychol. 2021 Oct 29;12:730985. doi: 10.3389/fpsyg.2021.730985 (PMC8586539; doi:10.3389/fpsyg.2021.730985)
Supplement: Supplementary Table 5 — Exploratory Analyses for Reasoning Data.docx. [file Table_5.docx]

**Exploratory Analyses for Reasoning Data**

Tables 1–14 report the GLMMs with a given linguistic feature as the dependent variable (DV).

| **Table 1.** *Comparison* as DV |  |  |  |  |  |
| --- | --- | --- | --- | --- | --- |
| Random Effects | Variance | *SD* |  |  |  |
| Participant (Intercept) | 0.033 | 0.182 |  |  |  |
| Description (Intercept) | 0.006 | 0.077 |  |  |  |
| Actor (Intercept) | 0.003 | 0.055 |  |  |  |
|  |  |  |  |  |  |
| Fixed Effects | Estimate | *SE* | *df* | *t* | *p* |
| (Intercept) | 0.060 | 0.048 | 119.713 | 1.247 | .215 |
| Type (Non-pretender) | 0.054 | 0.075 | 146.011 | 0.728 | .468 |
| Condition (Identify) | −0.086 | 0.074 | 143.466 | −1.158 | .249 |
| Group (O&M) | −0.144 | 0.074 | 207.353 | −1.956 | .052 |
| Group (Sighted) | −0.064 | 0.051 | 200.478 | −1.243 | .215 |
| Type (Non-pretender) × Condition (Identify) | 0.664 | 0.159 | 95.419 | 4.179 | <.001* |
| Type (Non-pretender) × Group (O&M) | −0.249 | 0.117 | 3530.918 | −2.128 | .033* |
| Type (Non-pretender) × Group (Sighted) | −0.134 | 0.081 | 3541.972 | −1.656 | .098 |
| Condition (Identify) × Group (O&M) | −0.176 | 0.117 | 3502.424 | −1.507 | .132 |
| Condition (Identify) × Group (Sighted) | −0.010 | 0.081 | 3485.010 | −0.116 | .899 |
| Type (Non-pretender) × Condition (Identify) × Group (O&M) | −0.361 | 0.233 | 3491.348 | −1.550 | .121 |
| Type (Non-pretender) × Condition (Identify) × Group (Sighted) | −0.509 | 0.161 | 3477.494 | −3.159 | .002* |

* *p* < .05

| **Table 2.** *Cognitive Processes* as DV |  |  |  |  |  |
| --- | --- | --- | --- | --- | --- |
| Random Effects | Variance | *SD* |  |  |  |
| Participant (Intercept) | 0.097 | 0.311 |  |  |  |
| Description (Intercept) | 0.004 | 0.062 |  |  |  |
| Actor (Intercept) | 0.004 | 0.000 |  |  |  |
|  |  |  |  |  |  |
| Fixed Effects | Estimate | *SE* | *df* | *t* | *p* |
| (Intercept) | −0.023 | 0.058 | 191.282 | −0.394 | .694 |
| Type (Non-pretender) | −0.209 | 0.071 | 301.801 | −2.921 | .004* |
| Condition (Identify) | 0.124 | 0.071 | 293.517 | 1.745 | .082 |
| Group (O&M) | −0.009 | 0.095 | 208.703 | −0.091 | .927 |
| Group (Sighted) | 0.046 | 0.067 | 197.381 | 0.695 | .488 |
| Type (Non-pretender) × Condition (Identify) | 0.058 | 0.142 | 292.821 | 0.408 | .684 |
| Type (Non-pretender) × Group (O&M) | 0.253 | 0.114 | 3484.552 | 2.215 | .027* |
| Type (Non-pretender) × Group (Sighted) | 0.074 | 0.079 | 3486.085 | 0.929 | .353 |
| Condition (Identify) × Group (O&M) | 0.069 | 0.114 | 3466.916 | 0.609 | .542 |
| Condition (Identify) × Group (Sighted) | 0.031 | 0.078 | 3440.943 | 0.395 | .693 |
| Type (Non-pretender) × Condition (Identify) × Group (O&M) | −0.007 | 0.227 | 3446.667 | −0.030 | .976 |
| Type (Non-pretender) × Condition (Identify) × Group (Sighted) | −0.259 | 0.157 | 3432.782 | −1.654 | .098 |

* *p* < .05

| **Table 3.** *Insight* as DV |  |  |  |  |  |
| --- | --- | --- | --- | --- | --- |
| Random Effects | Variance | *SD* |  |  |  |
| Participant (Intercept) | 0.098 | 0.313 |  |  |  |
| Description (Intercept) | 0.002 | 0.046 |  |  |  |
| Actor (Intercept) | 0.000 | 0.000 |  |  |  |
|  |  |  |  |  |  |
| Fixed Effects | Estimate | *SE* | *df* | *t* | *p* |
| (Intercept) | −0.121 | 0.058 | 191.400 | −2.090 | .038* |
| Type (Non-pretender) | −0.029 | 0.070 | 365.500 | −0.411 | .681 |
| Condition (Identify) | −0.022 | 0.069 | 355.500 | −0.315 | .753 |
| Group (O&M) | 0.067 | 0.096 | 209.800 | 0.696 | .487 |
| Group (Sighted) | 0.166 | 0.067 | 198.300 | 2.487 | .014* |
| Type (Non-pretender) × Condition (Identify) | 0.114 | 0.138 | 354.200 | 0.827 | .409 |
| Type (Non-pretender) × Group (O&M) | 0.134 | 0.114 | 3487.000 | 1.175 | .240 |
| Type (Non-pretender) × Group (Sighted) | 0.107 | 0.079 | 3483.000 | 1.353 | .176 |
| Condition (Identify) × Group (O&M) | 0.242 | 0.114 | 3470.000 | 2.126 | .034* |
| Condition (Identify) × Group (Sighted) | 0.190 | 0.078 | 3438.000 | 2.429 | .015* |
| Type (Non-pretender) × Condition (Identify) × Group (O&M) | 0.292 | 0.227 | 3449.000 | 1.285 | .199 |
| Type (Non-pretender) × Condition (Identify) × Group (Sighted) | 0.002 | 0.157 | 3430.000 | 0.012 | .991 |

* *p* < .05

| **Table 4.** *Causation* as DV |  |  |  |  |  |
| --- | --- | --- | --- | --- | --- |
| Random Effects | Variance | *SD* |  |  |  |
| Participant (Intercept) | 0.097 | 0.312 |  |  |  |
| Description (Intercept) | 0.004 | 0.061 |  |  |  |
| Actor (Intercept) | 0.000 | 0.000 |  |  |  |
|  |  |  |  |  |  |
| Fixed Effects | Estimate | *SE* | *df* | *t* | *p* |
| (Intercept) | 0.001 | 0.058 | 168.900 | 0.015 | .988 |
| Type (Non-pretender) | −0.056 | 0.071 | 317.400 | −0.791 | .429 |
| Condition (Identify) | 0.113 | 0.071 | 308.600 | 1.602 | .110 |
| Group (O&M) | 0.031 | 0.095 | 181.200 | 0.329 | .743 |
| Group (Sighted) | −0.011 | 0.067 | 171.300 | −0.158 | .875 |
| Type (Non-pretender) × Condition (Identify) | 0.199 | 0.142 | 307.900 | 1.407 | .160 |
| Type (Non-pretender) × Group (O&M) | 0.436 | 0.114 | 3474.000 | 3.807 | <.001* |
| Type (Non-pretender) × Group (Sighted) | 0.154 | 0.079 | 3472.000 | 1.952 | .051 |
| Condition (Identify) × Group (O&M) | 0.167 | 0.114 | 3454.000 | 1.467 | .142 |
| Condition (Identify) × Group (Sighted) | 0.063 | 0.078 | 3420.000 | 0.804 | .422 |
| Type (Non-pretender) × Condition (Identify) × Group (O&M) | 0.171 | 0.227 | 3430.000 | 0.750 | .453 |
| Type (Non-pretender) × Condition (Identify) × Group (Sighted) | 0.011 | 0.157 | 3410.000 | 0.071 | .943 |

* *p* < .05

| **Table 5.** *Tentativeness* as DV |  |  |  |  |  |
| --- | --- | --- | --- | --- | --- |
| Random Effects | Variance | *SD* |  |  |  |
| Participant (Intercept) | 0.089 | 0.298 |  |  |  |
| Description (Intercept) | 0.000 | 0.000 |  |  |  |
| Actor (Intercept) | 0.000 | 0.000 |  |  |  |
|  |  |  |  |  |  |
| Fixed Effects | Estimate | *SE* | *df* | *t* | *p* |
| (Intercept) | −0.069 | 0.056 | 191.969 | −1.243 | .215 |
| Type (Non-pretender) | −0.172 | 0.068 | 3494.552 | −2.526 | .012* |
| Condition (Identify) | −0.050 | 0.067 | 3435.941 | −0.735 | .463 |
| Group (O&M) | −0.054 | 0.093 | 205.592 | −0.578 | .564 |
| Group (Sighted) | 0.135 | 0.065 | 194.283 | 2.076 | .039* |
| Type (Non-pretender) × Condition (Identify) | −0.232 | 0.135 | 3437.651 | −1.723 | .085 |
| Type (Non-pretender) × Group (O&M) | 0.047 | 0.115 | 3507.650 | 0.411 | .681 |
| Type (Non-pretender) × Group (Sighted) | 0.055 | 0.079 | 3490.328 | 0.694 | .488 |
| Condition (Identify) × Group (O&M) | −0.132 | 0.114 | 3488.234 | −1.159 | .247 |
| Condition (Identify) × Group (Sighted) | −0.005 | 0.079 | 3442.278 | −0.065 | .949 |
| Type (Non-pretender) × Condition (Identify) × Group (O&M) | 0.201 | 0.228 | 3467.393 | 0.880 | .379 |
| Type (Non-pretender) × Condition (Identify) × Group (Sighted) | −0.062 | 0.157 | 3433.898 | −0.395 | .693 |

* *p* < .05

| **Table 6.** *Discrepancy* as DV |  |  |  |  |  |
| --- | --- | --- | --- | --- | --- |
| Random Effects | Variance | *SD* |  |  |  |
| Participant (Intercept) | 0.059 | 0.243 |  |  |  |
| Description (Intercept) | 0.001 | 0.024 |  |  |  |
| Actor (Intercept) | 0.001 | 0.037 |  |  |  |
|  |  |  |  |  |  |
| Fixed Effects | Estimate | *SE* | *df* | *t* | *p* |
| (Intercept) | −0.017 | 0.051 | 173.077 | −0.327 | .744 |
| Type (Non-pretender) | −0.131 | 0.069 | 237.928 | −1.886 | .061 |
| Condition (Identify) | −0.127 | 0.069 | 231.917 | −1.852 | .065 |
| Group (O&M) | −0.090 | 0.083 | 217.482 | −1.083 | .280 |
| Group (Sighted) | 0.060 | 0.058 | 207.286 | 1.040 | .300 |
| Type (Non-pretender) × Condition (Identify) | −0.062 | 0.143 | 185.538 | −0.435 | .664 |
| Type (Non-pretender) × Group (O&M) | 0.140 | 0.116 | 3515.473 | 1.206 | .228 |
| Type (Non-pretender) × Group (Sighted) | −0.044 | 0.080 | 3514.959 | −0.552 | .581 |
| Condition (Identify) × Group (O&M) | 0.021 | 0.116 | 3491.793 | 0.183 | .854 |
| Condition (Identify) × Group (Sighted) | 0.053 | 0.080 | 3463.116 | 0.669 | .504 |
| Type (Non-pretender) × Condition (Identify) × Group (O&M) | −0.250 | 0.231 | 3477.084 | −1.085 | .278 |
| Type (Non-pretender) × Condition (Identify) × Group (Sighted) | −0.325 | 0.159 | 3455.475 | −2.044 | .041* |

* *p* < .05

| **Table 7.** *Certainty* as DV |  |  |  |  |  |
| --- | --- | --- | --- | --- | --- |
| Random Effects | Variance | *SD* |  |  |  |
| Participant (Intercept) | 0.075 | 0.274 |  |  |  |
| Description (Intercept) | 0.000 | 0.000 |  |  |  |
| Actor (Intercept) | 0.000 | 0.000 |  |  |  |
|  |  |  |  |  |  |
| Fixed Effects | Estimate | *SE* | *df* | *t* | *p* |
| (Intercept) | 0.073 | 0.053 | 204.110 | 1.376 | .170 |
| Type (Non-pretender) | 0.154 | 0.068 | 3509.695 | 2.257 | .024* |
| Condition (Identify) | 0.356 | 0.068 | 3451.318 | 5.251 | <.001* |
| Group (O&M) | −0.028 | 0.089 | 217.955 | −0.313 | .755 |
| Group (Sighted) | −0.132 | 0.062 | 206.599 | −2.132 | .034* |
| Type (Non-pretender) × Condition (Identify) | 0.194 | 0.136 | 3453.341 | 1.428 | .153 |
| Type (Non-pretender) × Group (O&M) | −0.023 | 0.115 | 3520.596 | −0.197 | .844 |
| Type (Non-pretender) × Group (Sighted) | −0.095 | 0.080 | 3505.317 | −1.192 | .233 |
| Condition (Identify) × Group (O&M) | −0.381 | 0.115 | 3499.969 | −3.318 | .001* |
| Condition (Identify) × Group (Sighted) | −0.323 | 0.079 | 3457.159 | −4.084 | <.001* |
| Type (Non-pretender) × Condition (Identify) × Group (O&M) | −0.283 | 0.229 | 3481.441 | −1.234 | .217 |
| Type (Non-pretender) × Condition (Identify) × Group (Sighted) | −0.373 | 0.158 | 3449.210 | −2.361 | .018* |

* *p* < .05

| **Table 8.** *Differentiation* as DV |  |  |  |  |  |
| --- | --- | --- | --- | --- | --- |
| Random Effects | Variance | *SD* |  |  |  |
| Participant (Intercept) | 0.038 | 0.194 |  |  |  |
| Description (Intercept) | 0.003 | 0.059 |  |  |  |
| Actor (Intercept) | 0.003 | 0.056 |  |  |  |
|  |  |  |  |  |  |
| Fixed Effects | Estimate | *SE* | *df* | *t* | *p* |
| (Intercept) | 0.021 | 0.048 | 103.300 | 0.433 | .666 |
| Type (Non-pretender) | −0.284 | 0.072 | 170.200 | −3.936 | <.001* |
| Condition (Identify) | −0.070 | 0.072 | 166.700 | −0.980 | .329 |
| Group (O&M) | 0.029 | 0.075 | 209.300 | 0.389 | .698 |
| Group (Sighted) | 0.010 | 0.052 | 201.700 | 0.190 | .850 |
| Type (Non-pretender) × Condition (Identify) | −0.307 | 0.154 | 68.340 | −1.993 | .050 |
| Type (Non-pretender) × Group (O&M) | −0.062 | 0.116 | 3523.000 | −0.537 | .591 |
| Type (Non-pretender) × Group (Sighted) | −0.039 | 0.080 | 3536.000 | −0.491 | .623 |
| Condition (Identify) × Group (O&M) | 0.043 | 0.115 | 3495.000 | 0.373 | .709 |
| Condition (Identify) × Group (Sighted) | 0.126 | 0.080 | 3480.000 | 1.580 | .114 |
| Type (Non-pretender) × Condition (Identify) × Group (O&M) | −0.195 | 0.231 | 3482.000 | −0.844 | .398 |
| Type (Non-pretender) × Condition (Identify) × Group (Sighted) | 0.028 | 0.160 | 3472.000 | 0.174 | .862 |

* *p* < .05

| **Table 9.** *Perceptual Processes* as DV |  |  |  |  |  |
| --- | --- | --- | --- | --- | --- |
| Random Effects | Variance | *SD* |  |  |  |
| Participant (Intercept) | 0.170 | 0.412 |  |  |  |
| Description (Intercept) | 0.008 | 0.091 |  |  |  |
| Actor (Intercept) | 0.007 | 0.086 |  |  |  |
|  |  |  |  |  |  |
| Fixed Effects | Estimate | *SE* | *df* | *t* | *p* |
| (Intercept) | 0.003 | 0.074 | 176.500 | 0.045 | .964 |
| Type (Non-pretender) | 0.038 | 0.072 | 111.700 | 0.521 | .604 |
| Condition (Identify) | −0.352 | 0.072 | 107.700 | −4.905 | <.001* |
| Group (O&M) | −0.114 | 0.114 | 214.500 | −0.996 | .320 |
| Group (Sighted) | 0.015 | 0.080 | 201.300 | 0.191 | .849 |
| Type (Non-pretender) × Condition (Identify) | −0.164 | 0.168 | 53.320 | −0.982 | .331 |
| Type (Non-pretender) × Group (O&M) | −0.059 | 0.109 | 3444.000 | −0.546 | .585 |
| Type (Non-pretender) × Group (Sighted) | −0.022 | 0.075 | 3444.000 | −0.296 | .767 |
| Condition (Identify) × Group (O&M) | 0.052 | 0.108 | 3438.000 | 0.482 | .630 |
| Condition (Identify) × Group (Sighted) | 0.228 | 0.074 | 3414.000 | 3.060 | .002* |
| Type (Non-pretender) × Condition (Identify) × Group (O&M) | −0.002 | 0.215 | 3417.000 | −0.011 | .991 |
| Type (Non-pretender) × Condition (Identify) × Group (Sighted) | −0.163 | 0.149 | 3407.000 | −1.093 | .274 |

* *p* < .05

| **Table 10.** *See* as DV |  |  |  |  |  |
| --- | --- | --- | --- | --- | --- |
| Random Effects | Variance | *SD* |  |  |  |
| Participant (Intercept) | 0.094 | 0.307 |  |  |  |
| Description (Intercept) | 0.023 | 0.152 |  |  |  |
| Actor (Intercept) | 0.018 | 0.133 |  |  |  |
|  |  |  |  |  |  |
| Fixed Effects | Estimate | *SE* | *df* | *t* | *p* |
| (Intercept) | 0.048 | 0.070 | 77.900 | 0.681 | .498 |
| Type (Non-pretender) | −0.022 | 0.084 | 56.970 | −0.262 | .794 |
| Condition (Identify) | −0.658 | 0.083 | 55.630 | −7.894 | <.001* |
| Group (O&M) | −0.154 | 0.092 | 198.800 | −1.664 | .098 |
| Group (Sighted) | −0.005 | 0.065 | 188.500 | −0.078 | .938 |
| Type (Non-pretender) × Condition (Identify) | −0.130 | 0.213 | 30.930 | −0.608 | .547 |
| Type (Non-pretender) × Group (O&M) | −0.034 | 0.108 | 3468.000 | −0.314 | .754 |
| Type (Non-pretender) × Group (Sighted) | −0.104 | 0.075 | 3462.000 | −1.393 | .164 |
| Condition (Identify) × Group (O&M) | 0.003 | 0.107 | 3451.000 | 0.025 | .980 |
| Condition (Identify) × Group (Sighted) | 0.257 | 0.074 | 3419.000 | 3.459 | .001* |
| Type (Non-pretender) × Condition (Identify) × Group (O&M) | 0.104 | 0.214 | 3430.000 | 0.485 | .628 |
| Type (Non-pretender) × Condition (Identify) × Group (Sighted) | −0.270 | 0.148 | 3411.000 | −1.823 | .068 |

* *p* < .05

| **Table 11.** *Feel* as DV |  |  |  |  |  |
| --- | --- | --- | --- | --- | --- |
| Random Effects | Variance | *SD* |  |  |  |
| Participant (Intercept) | 0.077 | 0.277 |  |  |  |
| Description (Intercept) | 0.000 | 0.000 |  |  |  |
| Actor (Intercept) | 0.002 | 0.047 |  |  |  |
|  |  |  |  |  |  |
| Fixed Effects | Estimate | *SE* | *df* | *t* | *p* |
| (Intercept) | −0.095 | 0.055 | 172.890 | −1.739 | .084 |
| Type (Non-pretender) | −0.111 | 0.069 | 3491.663 | −1.611 | .107 |
| Condition (Identify) | 0.109 | 0.068 | 3432.647 | 1.602 | .109 |
| Group (O&M) | −0.011 | 0.089 | 205.866 | −0.128 | .898 |
| Group (Sighted) | 0.154 | 0.062 | 195.359 | 2.479 | .014* |
| Type (Non-pretender) × Condition (Identify) | −0.123 | 0.144 | 168.177 | −0.855 | .394 |
| Type (Non-pretender) × Group (O&M) | 0.040 | 0.116 | 3509.926 | 0.346 | .729 |
| Type (Non-pretender) × Group (Sighted) | 0.153 | 0.080 | 3500.389 | 1.910 | .056 |
| Condition (Identify) × Group (O&M) | −0.115 | 0.115 | 3488.183 | −0.995 | .320 |
| Condition (Identify) × Group (Sighted) | 0.051 | 0.079 | 3450.006 | 0.647 | .518 |
| Type (Non-pretender) × Condition (Identify) × Group (O&M) | −0.164 | 0.230 | 3465.097 | −0.716 | .474 |
| Type (Non-pretender) × Condition (Identify) × Group (Sighted) | 0.209 | 0.158 | 3441.462 | 1.321 | .187 |

* *p* < .05

| **Table 12.** *Hear* as DV |  |  |  |  |  |
| --- | --- | --- | --- | --- | --- |
| Random Effects | Variance | *SD* |  |  |  |
| Participant (Intercept) | 0.108 | 0.328 |  |  |  |
| Description (Intercept) | 0.014 | 0.118 |  |  |  |
| Actor (Intercept) | 0.000 | 0.003 |  |  |  |
|  |  |  |  |  |  |
| Fixed Effects | Estimate | *SE* | *df* | *t* | *p* |
| (Intercept) | 0.059 | 0.063 | 195.438 | 0.932 | .353 |
| Type (Non-pretender) | 0.094 | 0.079 | 117.970 | 1.187 | .238 |
| Condition (Identify) | 0.136 | 0.079 | 114.782 | 1.731 | .086 |
| Group (O&M) | −0.060 | 0.098 | 225.113 | −0.608 | .544 |
| Group (Sighted) | −0.101 | 0.069 | 212.983 | −1.472 | .143 |
| Type (Non-pretender) × Condition (Identify) | −0.087 | 0.158 | 116.770 | −0.555 | .580 |
| Type (Non-pretender) × Group (O&M) | −0.146 | 0.113 | 3475.470 | −1.297 | .195 |
| Type (Non-pretender) × Group (Sighted) | −0.090 | 0.078 | 3480.783 | −1.157 | .247 |
| Condition (Identify) × Group (O&M) | 0.066 | 0.112 | 3461.295 | 0.591 | .555 |
| Condition (Identify) × Group (Sighted) | 0.070 | 0.078 | 3442.578 | 0.905 | .365 |
| Type (Non-pretender) × Condition (Identify) × Group (O&M) | −0.137 | 0.224 | 3441.800 | −0.612 | .540 |
| Type (Non-pretender) × Condition (Identify) × Group (Sighted) | 0.091 | 0.155 | 3435.760 | 0.585 | .559 |

| **Table 13.** *Body* as DV |  |  |  |  |  |
| --- | --- | --- | --- | --- | --- |
| Random Effects | Variance | *SD* |  |  |  |
| Participant (Intercept) | 0.010 | 0.100 |  |  |  |
| Description (Intercept) | 0.043 | 0.208 |  |  |  |
| Actor (Intercept) | 0.000 | 0.001 |  |  |  |
|  |  |  |  |  |  |
| Fixed Effects | Estimate | *SE* | *df* | *t* | *p* |
| (Intercept) | −0.031 | 0.053 | 76.655 | −0.581 | .563 |
| Type (Non-pretender) | −0.072 | 0.101 | 72.367 | −0.711 | .480 |
| Condition (Identify) | −0.025 | 0.101 | 72.067 | −0.246 | .806 |
| Group (O&M) | 0.029 | 0.063 | 203.594 | 0.457 | .648 |
| Group (Sighted) | 0.048 | 0.044 | 201.274 | 1.106 | .270 |
| Type (Non-pretender) × Condition (Identify) | 0.303 | 0.202 | 72.095 | 1.498 | .139 |
| Type (Non-pretender) × Group (O&M) | 0.019 | 0.116 | 3541.814 | 0.160 | .873 |
| Type (Non-pretender) × Group (Sighted) | 0.069 | 0.081 | 3551.448 | 0.858 | .391 |
| Condition (Identify) × Group (O&M) | −0.071 | 0.116 | 3515.511 | −0.609 | .543 |
| Condition (Identify) × Group (Sighted) | 0.059 | 0.080 | 3498.714 | 0.735 | .462 |
| Type (Non-pretender) × Condition (Identify) × Group (O&M) | −0.366 | 0.232 | 3510.149 | −1.577 | .115 |
| Type (Non-pretender) × Condition (Identify) × Group (Sighted) | 0.219 | 0.161 | 3494.303 | 1.362 | .173 |

| **Table 14.** *Space* as DV |  |  |  |  |  |
| --- | --- | --- | --- | --- | --- |
| Random Effects | Variance | *SD* |  |  |  |
| Participant (Intercept) | 0.097 | 0.311 |  |  |  |
| Description (Intercept) | 0.015 | 0.120 |  |  |  |
| Actor (Intercept) | 0.004 | 0.067 |  |  |  |
|  |  |  |  |  |  |
| Fixed Effects | Estimate | *SE* | *df* | *t* | *p* |
| (Intercept) | 0.100 | 0.063 | 136.176 | 1.570 | .119 |
| Type (Non-pretender) | 0.050 | 0.080 | 79.465 | 0.623 | .535 |
| Condition (Identify) | 0.013 | 0.080 | 77.337 | 0.161 | .873 |
| Group (O&M) | −0.110 | 0.095 | 197.122 | −1.158 | .248 |
| Group (Sighted) | −0.111 | 0.067 | 186.979 | −1.674 | .096 |
| Type (Non-pretender) × Condition (Identify) | −0.346 | 0.173 | 58.497 | −2.000 | .050 |
| Type (Non-pretender) × Group (O&M) | 0.048 | 0.114 | 3473.730 | 0.419 | .676 |
| Type (Non-pretender) × Group (Sighted) | −0.093 | 0.079 | 3474.631 | −1.173 | .241 |
| Condition (Identify) × Group (O&M) | 0.032 | 0.114 | 3455.488 | 0.278 | .781 |
| Condition (Identify) × Group (Sighted) | 0.049 | 0.078 | 3428.495 | 0.622 | .534 |
| Type (Non-pretender) × Condition (Identify) × Group (O&M) | 0.444 | 0.227 | 3434.646 | 1.960 | .050 |
| Type (Non-pretender) × Condition (Identify) × Group (Sighted) | 0.437 | 0.157 | 3420.365 | 2.787 | .005* |

* *p* < .05

**Assessing why greater *comparisons* were found for pretender descriptions rather than non-pretender descriptions:**

In the *Chance* condition, blind, sighted, and O&M judges’ reasoning contained more *comparison* terms in pretender descriptions than non-pretender descriptions. Because we did not have specific hypotheses, we further explored the reasoning data in an attempt to better understand why greater *comparisons* were found for pretender descriptions rather than non-pretender descriptions. Here, we provide one possible interpretation: The judges’ reasoning data in non-pretender descriptions suggested that non-pretender descriptions conveyed specific information which made it relatively apparent that the individual was sighted. As a result, the judges seemed to simply point to this information as their rationale. In other words, it is possible that the non-pretender descriptions in the *Chance* condition did not provide as many opportunities for judges to elaborate and make comparisons in their rationale compared to the pretender descriptions.

As an example, two descriptions in the *Chance* condition mentioned “j-walking”:

*I usually just press the button and then I will wait for the signal and watch for it to actually have the countdown for me to be safely walk over it. But depending on the intersections, busy or not, I do sometimes j-walk and just walk across when I notice it is not busy or there is no cars on the road.*

*As I am approaching it, I am already looking at the signal to see if it started to count down. If so, I know I will probably be crossing the opposite direction by the time I get there. If it hasn’t started to count down yet, then that means I have enough time to get to the intersection and cross that direction before the light will turn red. So, I can kind of speed up my walking if that is the case. If the traffic is very light, for example in the very early morning, I sometimes j-walk a little bit. Because I can see that there are no cars coming. So, I do not have to wait then for the crosswalk to be clear.*

Several judges who correctly identified these descriptions as coming from a non-pretender simply stated that j-walking was mentioned in their rationale. For example: “*use of the j walking term*”, “*they state they j walk if no traffic*”, “*they j walk*”, “*they talked about j walking*”. In other words, j-walking is an activity that sighted—but not blind—individuals tend to engage in which suggested that the description likely came from an individual who was actually sighted (non-pretender). Other descriptions also elicited similar reasoning that were short and pointed to specific aspects of the description.

If non-pretender descriptions in the *Chance* condition did not provide as many opportunities for judges to elaborate and make comparisons in their rationale compared to the pretender descriptions, this would suggest that reasoning data in non-pretender responses should be shorter than reasoning data in pretender responses. To test this, we ran a GLMM with word count as the dependent variable, *Group* (blind, sighted, O&M) and *Response* (pretender, non-pretender) as fixed effects, and *Subject* (i.e., 221 judges) and *Item* (i.e., 32 trials) as random effects. Only responses in the *Chance* condition were included in this analysis. Reasoning data for the non-pretender descriptions contained a significantly lower word count than reasoning data in pretender descriptions, supporting our hypothesis (Table 15). This interpretation might imply that sighted people’s descriptions of crossing the road with vision (non-pretenders) better conveyed their sightedness than blind people’s descriptions of crossing the road with vision. Because these are exploratory analyses, follow-up analyses should be conducted to examine the validity of this interpretation.

| ***Table 15.*** *Word Count* as DV |  |  |  |  |  |
| --- | --- | --- | --- | --- | --- |
| Random Effects | Variance | *SD* |  |  |  |
| Participant (Intercept) | 0.466 | 0.683 |  |  |  |
| Description (Intercept) | 0.019 | 0.139 |  |  |  |
| Actor (Intercept) | 0.000 | 0.000 |  |  |  |
|  |  |  |  |  |  |
| Fixed Effects | Estimate | *SE* | *df* | *t* | *p* |
| (Intercept) | 0.126 | 0.108 | 214.842 | 1.171 | .243 |
| Type (Non-pretender) | −0.203 | 0.071 | 91.029 | −2.871 | .005* |
| Group (O&M) | −0.256 | 0.169 | 212.322 | −1.520 | .130 |
| Group (Sighted) | −0.172 | 0.121 | 199.593 | −1.420 | .157 |
| Type (Non-pretender) × Group (O&M) | 0.049 | 0.086 | 3382.663 | 0.570 | .569 |
| Type (Non-pretender) × Group (Sighted) | 0.108 | 0.059 | 3377.982 | 1.821 | .069 |

* *p* < .05
