# Supplementary material for: The Modified Imitation Game: A Method for Measuring Interactional Expertise
Source: Front Psychol. 2021 Oct 29;12:730985. doi: 10.3389/fpsyg.2021.730985 (PMC8586539; doi:10.3389/fpsyg.2021.730985)
Supplement: Supplementary Presentation 1 — Description of Supplementary Material files. [file Presentation_1.pdf]

Description of Supplementary Material files

	File name	Description
1.	Demographic_Phase_I.csv	This file is not cited in the text. This is the demographic data for Phase I participants. This file also includes the descriptions elicited from the actors (i.e., Phase I participants).
2.	Demographic_Phase_II.csv	This file is not cited in the text. This is the demographic data for Phase 2 participants.
3.	Instructions for Procedure - Phase II.docx	These are the instructions that were included in the Qualtrics survey (Phase II).
4.	Reproducible Code for Judges Decision.docx	This is Rmarkdown code for running the analyses for judges' decisions. Frontiers does not allow Rmd files to be uploaded. Instructions for converting the Word document to Rmd are included on the first page.
5.	Judges_Decision_Data.csv	Judges' decision data.
6.	Principal Components Analysis.docx	Results of the Principal Components Analysis (PCA) that was performed on the random effects to determine the optimal random-effects structure using the rePCA() function in the lme4 package.
7.	Reproducible Code for Judges Reasoning.docx	This is Rmarkdown code for running the analyses for judges' reasoning. Frontiers does not allow Rmd files to be uploaded. Instructions for converting the Word document to Rmd are included on the first page.
8.	Judges_Reasoning_Data.csv	Judges' reasoning data that were analyzed using LIWC.
9.	Exploratory Analyses for Reasoning Data.docx	The 14 linguistic features were entered as the dependent variable in separate GLMMs, with each model predicting a separate linguistic feature. This document contains the results of these exploratory analyses.
